# Four New Furostanol Saponins from the Rhizomes and Roots of *Smilax scobinicaulis* and Their Cytotoxicity

**DOI:** 10.3390/molecules191220975

**Published:** 2014-12-15

**Authors:** Jing Xu, Shixiu Feng, Qi Wang, Yingli Cao, Miao Sun, Cunli Zhang

**Affiliations:** 1 College of Life Science, Northwest A&F University, Yangling 712100, Shaanxi, China; E-Mails: seaqisky@163.com (J.X.); wangqidream2006@163.com (Q.W.); caoyinglisky@163.com (Y.C.); sunmiao151@163.com (M.S.); 2 Key Laboratory of Southern Subtropical Plant Diversity, Shenzhen Fairy Lake Botanical Garden, Chinese Academy of Sciences, 160 Xianhu Road, Liantang, Shenzhen 518004, China; E-Mail: shixiufeng7787@gmail.com; 3 Shenzhen Boton Flavors & Fragrances Co., Ltd., 19 Daxin Road, Nanshan, Shenzhen 518051, China

**Keywords:** *Smilax scobinicaulis*, furostanol saponins, cytotoxic activity

## Abstract

Four new furostanol saponins **1**–**4**, along with two known furostanol saponins **5** and **6** and one known spirostanol saponin **7** were isolated from the rhizomes and roots of *Smilax scobinicaulis*. The structures of the new saponins were elucidated as 26-*O*-β-d-glucopyranoside-3β,26-dihydroxy-(25*R*)-5α-furostan-22-methoxyl-6-one-3-*O*-α-l-arabinopyranosyl-(1→6)-β-d-glucopyranoside (**1**), 26-*O*-β-d-glucopyranoside-3β,26-dihydroxy-(25*R*)-5α-furostan-22-methoxyl-6-one (**2**), 26-*O*-β-d-glucopyranoside-3β,26-dihydroxy-(25*R*)-5α-furostan-20(22)-en-6-one (**3**), 26-*O*-β-d-glucopyranoside-3β,23,26-trihydroxy-(23*R*, 25*R*)-5α-furostan-20(22)-en-6-one (**4**) on the basis of spectroscopic analysis. The isolated saponins were evaluated for cytotoxic activity against two human cancer cell lines including Hela (cervical carcinoma) and SMMC-7221 (hepatocellular carcinoma). Compounds **1** and **7** demonstrated cytotoxicity against the tested cell lines.

## 1. Introduction

The genus *Smilax* (family Liliaceae) is mainly distributed in the tropical, subtropical and temperate areas of the world [1]. It is well known that steroidal saponins are abundant in the genus *Smilax* [2]. Many species of this genus have a long history of use as herbal remedies. *Smilax scobinicaulis* (C.H.) Wright, commonly known as Hei Ci Ba Qia in Chinese is one of them, which is distributed in Shaanxi, Gansu, Sichuan, Yunnan and other regions of China [3]. The rhizomes and roots of this plant, known as “Jin Gang Teng” and “Wei Ling Xian” in North China, have long been used in folk medicine for the treatment of rheumatic arthritis, lumbago, gout, tumor and inflammatory diseases [1]. Previous phytochemical investigations of this plant led to the isolation of steroidal saponins [4], flavonoids [5] and phenylpropanoids [6]. Bioactivity investigations showed that some of the isolated steroidal saponins had antimicrobial and cytotoxic activity [4,7].

**Figure 1:**
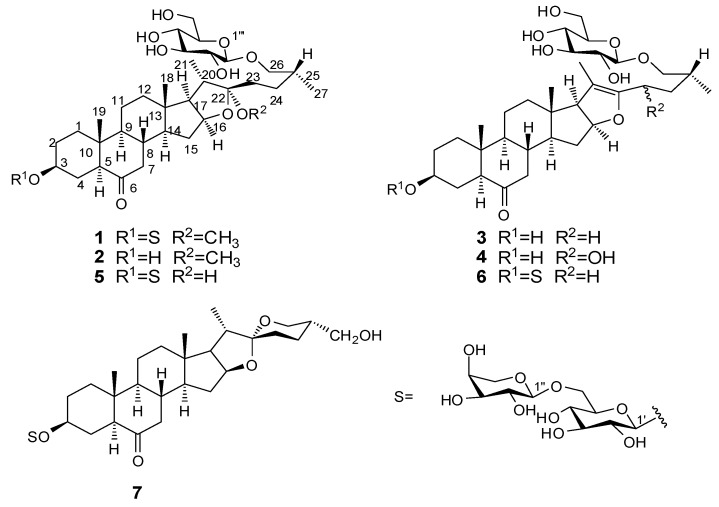
Chemical structure of compounds **1**–**7**.

In a continuing search for new steroidal saponins from this plant, a series of steroidal saponins have now been obtained, including four new furostanol saponins **1**–**4**, two known furostanol saponins **5** and **6** and one known spirostanol saponin **7** (Figure 1), which are reported here as *S. scobinicaulis* compounds for the first time. All the compounds have been tested for cytotoxicity against Hela and SMMC-7221 human cancer cell lines. In this paper, we report the isolation and cytotoxic activity of these compounds and the structural elucidation of the new compounds.

## 2. Results and Discussion

The rhizomes and roots of *S. scobinicaulis* were extracted with ethanol (EtOH). The extract was suspended in water (H_2_O) and partitioned successively with petroleum ether (PE), ethyl acetate (EtOAc) and *n-*butanol (*n*-BuOH). The *n*-BuOH soluble fraction was subjected to silica gel CC (column chromatography), Sephadex LH-20, reversed phase silica gel CC and semi-preparative High Performance Liquid Chromatography (HPLC) to afford four new furostanol saponins 26-*O*-β-d-glucopyranoside-3β,26-dihydroxy-(25*R*)-5α-furostan-22-methoxyl-6-one-3-*O*-α-l-arabinopyranosyl-(1→6)-β-d-glucopyranoside (**1**), 26-*O*-β-d-glucopyranoside-3β,26-dihydroxy-(25*R*)-5α-furostan-22-methoxyl-6-one (**2**), 26-*O*-β-d-glucopyranoside-3β,26-dihydroxy-(25*R*)-5α-furostan-20(22)-en-6-one (**3**), 26-*O*-β-d-glucopyranoside-3β,23,26-trihydroxy-(23*R*, 25*R*)-5α-furostan-20(22)-en-6-one (**4**), along with two known furostanol saponins 26-*O*-β-d-glucopyranosyl-3β,22*ξ*,26-trihydroxy-(25*R*)-5α-furostan-6-one-3-*O*-α-l-arabinopyranosyl-(1→6)-β-d-glucopyranoside (**5**) [8], 26-*O*-β-d-gluco- pyranosyl-3β,26-dihydroxy-(25*R*)-5α-furostan-20(22)-en-6-one-3-*O*-α-l-arabinopyranosyl-(1→6)-β-d-glucopyranoside (**6**) [9] and the known spirostanol saponin sieboldogenin-3-*O*-α-l-arabino-pyranosyl-(1→6)-β-d-glucopyranoside (**7**) [10].

### 2.1. Structure Elucidation

**Figure 2:**
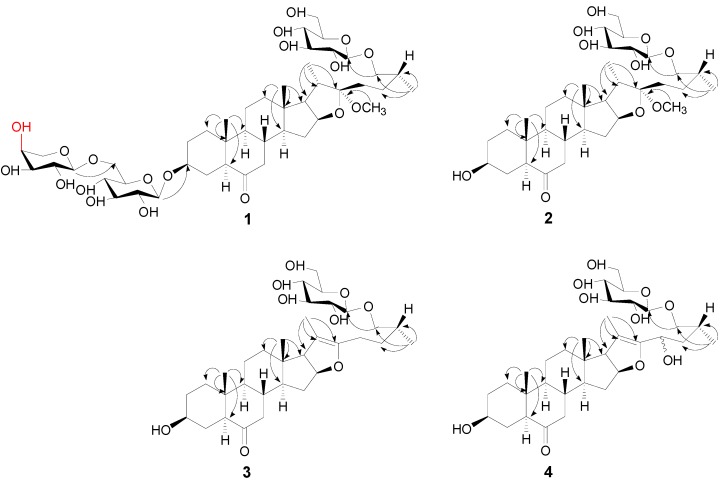
Key HMBC correlations of compounds **1**–**4**.

Compound **1** was isolated as a white amorphous powder and showed a positive reaction (red colour) to the Ehrlich reagent. The molecular formula, C_45_H_74_O_19,_ was established from a positive molecular ion peak at *m/z* 941.4727 [M+Na]^+^ (calcd. for C_45_H_74_NaO_19_: 941.4717) in the HR-ESI-MS spectrum and supported by the ESI-MS (*m/z* 941.4 [M+Na]^+^ and *m/z* 917.3 [M−H]^−^) spectrum. Its IR spectrum displayed strong absorption bands for hydroxyl groups at 3408 cm^−1^, for carbonyl group at 1707 cm^−1^ and absorption bands of alkyl groups at 2927 cm^−1^. The ^1^H- and ^13^C-NMR assignments of **1** were based on the DEPT and 2D-NMR (COSY, HSQC, HMBC and NOESY) experiments and with the positive red colour reaction in Ehrlich’s test suggested **1** was a furostanol saponin. To be specific, The ^1^H-NMR spectrum of **1** showed four methyl proton signals including two tertiary methyl groups at δ_H_0.86 (3H, s, H-18) and 0.80 (3H, s, H-19) and two secondary methyl groups at δ_H_ 1.04 (3H, d, *J* = 6.0 Hz, H-21) and 0.98 (3H, d, *J* = 5.0 Hz, H-27), corresponding to C-atom signals at δ_C_ 15.5, 12.1, 14.7 and 15.9 in HSQC spectrum, typical steroid methyl signals. Besides, a signal for carbonyl at δ_C_ 212.0 was present in the ^13^C-NMR. A correlation signal between H-7 at δ_H_ 2.16 and C-6 at δ_C_ 212.0 was observed in the HMBC spectrum (Figure 2), which suggested the carbonyl is located at C-6. Moreover, a methoxyl group at δ_H_ 3.17 (3H, s, OCH_3_) was also observed in the ^1^H-NMR spectrum. In the HMBC spectrum, the correlations from OCH_3_ at δ_H_ 3.17 to C-22 at δ_C_ 112.6 to suggested the OCH_3_ was linked to the C-22 (Figure 2). The configurations of 1 were mainly determined by NOESY spectrum. A NOESY correlation signal between the H-5 proton at δ_H_ 2.40 and the H-9 proton at δ_H_ 1.40 was consistent with the 5α configuration. A NOESY cross-peak between H-5 (δ_H_ 2.40) and H-3 (δ_H_ 3.36) indicated that H-3 was α configuration. In addition, The NOE correlation between H-18 (δ_H_ 0.86, 3H, s) and H-20 (δ_H_ 2.21, 1H, m) suggested that the C-21 methyl group was α-configuration. Thus, the α-configurations of H-17 and the methoxy at C-22 position were unambiguously deduced based on the strong NOE correlations of H-21/H-17 and H-21/OCH_3_. The 14α configuration was further confirmed by the NOE correlations of H-14/H-16 and H-16/OCH_3_ [11]. However, the C-25 configuration of **1** was assigned as 25*R* based on the observed difference (∆_ab_ = δ_a_ − δ_b_ = 0.35) of the ^1^H-NMR chemical shifts of the H_2_-26 geminal protons, which was in agreement with that of 25*R* furostane-type steroidal saponins (∆_ab_ < 0.48 for 25*R*; ∆_ab_ > 0.57 for 25*S*) [12].

In addition, three anomeric proton signals at δ_H_ 4.42 (1H, d, *J* = 6.5 Hz, glc-1'), 4.34 (1H, d, *J* = 6.0 Hz, ara-1'') and 4.26 (1H, d, *J* = 7.0 Hz, glc-1''') existed in the ^1^H-NMR spectrum and the HSQC showed correlations with anomeric carbon signals at δ_C_ 100.9, 103.8 and 103.2, respectively, indicating the presence of three sugar moieties. Combined the ^1^H-NMR, ^13^C-NMR (including DEPT) and 2D-NMR (HSQC, HMBC and COSY), two glucopyranosyls and one arabinopyranosyl were found. The relative configurations of the two glucopyranose moieties were all assigned as β-configurations based on their coupling constants (*J* = 6.5 Hz, Glc-1'; *J* = 7.0 Hz, Glc-1''') of the anomeric protons. The relative configuration of the arabinopyranose moiety was determined as α-confirguration by the coupling constants (*J* = 6.0 Hz, Ara-1''). The sugars were determined to be d-glucose and l-arabinose by acid hydrolysis of **1** and then trimethylsilylation and GC analysis on a chiral column in comparison with an authentic sample. Furthermore, the location of the glucopyranosyl was found to be C-26 of the aglycon on the basis of a glycosylation shift of C-26 at δ_C_ 74.6 and the HMBC correlation peak between H-1''' of glucopyranosyl at δ_H_ 4.26 and C-26 of the aglycone at δ_C_ 74.6, which is a structural feature in plant furostanol saponins [11]. The attachments of sugar chain were deduced from the HMBC spectrum, in which the long range correlation between glc-H-1' at δ_H_ 4.42 with C-3 at δ_C_ 76.8 of the aglycone and ara-H-1'' at δ_H_ 4.34 with C-6' at δ_C_ 68.2 of the glucopyranosyl were observed (Figure 2), respectively. These results implied that the glucopyranosyl was linked to C-3 of the aglycone and the two sugars were the 1→6 linkage. Thus, the structure of **1** was elucidated as 26-*O*-β-d-glucopyranoside*-*3β,26*-*dihydroxy-(25R)-5α-furostan-22-methoxyl-6-one-3-*O*-α-l-arabinopyranosyl-(1→6)-β-d-glucopyranoside.

Compound **2**, obtained as a white amorphous powder, gave a positive red colour reaction in Ehrlich’s test. The HR-ESI-MS spectrum of **2** showed a positive ion [M+Na]^+^ at *m/z* 647.3762, consistent with the molecular formula C_34_H_56_O_10_ (calcd. for C_34_H_56_NaO_10_: 647.3766), which was supported by the ESI-MS (*m/z* 647.4 [M+Na]^+^ and *m/z* 623.4 [M–H]^−^) spectrum. In the IR spectrum, the glycosidic nature of **2** was shown by strong hydroxyl groups absorptions at 3423 cm^−1^, a carbonyl group at 1713 cm^−1^ and the alkyl groups at 2927, 2872 and 2853 cm^−1^ was also displayed in the IR spectrum. Compound **2** was suggested to be a furostanol glycoside with one sugar moiety by Ehrlich’s test and the ^1^H- and ^13^C-NMR assignments of **2** based on the DEPT and 2D-NMR (COSY, HSQC, HMBC and NOESY) experiments. The aglycone of **2** was deduced as the same as that of **1** by comparison the ^1^H- and ^13^C-NMR spectra of **2** with those of **1**. This was further confirmed by comparison Rf values of the aglycones obtained by acid hydrolysis of **1** and **2** on TLC. The only one sugar moiety was assigned to C-26 by a glycosylation shift of C-26 at δ_C_ 74.6 and the HMBC correlation between H-1 of the glucopyranosyl at δ_H_ 4.26 and C-26 at δ_C_ 74.6 (Figure 2). The relative configuration of the glucopyranose moiety was determined as β by the coupling constant (*J* = 8.0 Hz, Glc-1''') of the anomeric proton. The glucopyranose moiety was determined as d-glucose by acid hydrolysis of **2** and then trimethylsilylation and GC analysis on a chiral column in the same manner. The 25*R* configuration of **2** was deduced by the difference (∆_ab_ = 0.35) of the chemical shifts of the H_2_-26 geminal protons. Thus, the structure of **2** was elucidated as 26-*O*-β-d-glucopyranoside-3β,26-dihydroxy-(25*R*)-5α-furostan-22α-methoxyl-6-one.

Compound **3** was obtained as a white amorphous powder and positive ion HR-ESI-MS provided an [M+Na]^+^ ion *m/z* 615.3510, corresponding to a molecular formula of C_33_H_52_O_9_ (calcd. for C_33_H_52_NaO_9_: 615.3504), which was supported by *m/z* 615.3 [M+H] ^+^ and *m/z* 591.3 [M–H]^−^ in the ESI-MS spectrum. The IR spectrum showed the presence of hydroxyl groups at 3421 cm^−1^, a carbonyl group at 1697 cm^−1^ and alkyl groups at 2943 cm^−1^, 2915 cm^−1^and 2865 cm^−1^. It was apparent from the NMR spectroscopic data (^1^H, ^13^C, COSY, HSQC, HMBC and NOESY) of **3** that this compound differed from **2** only by the presence of the ∆^20,22^ double bond. The occurrence of a ∆^20,22^ double bond was confirmed from the HMBC spectrum which showed significant correlation peaks between the methyl proton signal of CH_3_-21 with the carbon resonances of C-20 (δ_C_ 103.6) and C-22 (δ_C_ 151.8) (Figure 2). The sugar moiety also was assigned as β-glucopyranose based on the coupling constant (*J* = 8.0 Hz, Glc-1''') of the anomeric proton. Its absolute configuration was also a d- as determined by acid hydrolysis of **3** and then trimethylsilylation and GC analysis on a chiral column. The α-configuration of H-17 was determined by the NOE correlations of H-14/H-17. The 25*R* configuration of **3** was assigned by the observed difference (∆_ab_ = δ_a_ − δ_b_ = 0.32) of the ^1^H-NMR chemical shifts of the H_2_-26 geminal protons (∆_ab_ < 0.48 for 25*R*; ∆_ab_ > 0.57 for 25*S*). Therefore, compound **3** was identified as 26-*O*-β-d-glucopyranoside-3β,26-dihydroxy-(25*R*)-5α-furostan-20(22)-en-6-one.

Compound **4**, a white amorphous powder, was assigned as C_33_H_52_O_10_ on the basis of its HR-ESI-MS data [M+Na]^+^ *m/z* 631.3450 (calcd. for C_33_H_52_NaO_10_: 631.3453) and its ESI-MS data *m/z* 631.55 [M+Na]^+^, 609.34 [M+H]^−^ and 1215.03 [2M−H]^−^. The IR spectrum showed absorptions for hydroxyl groups (3422 cm^−1^), carbonyl group (1708 cm^−1^) and alkyl groups (2934 cm^−1^ and 2870 cm^−1^). The 1D and 2D NMR spectroscopic data of **4** were in good agreement with those of **3** except for noticeable differences in the δ_H_-23 and δ_C_-23 of the aglycone. The sugar moiety also was determined as β-d-glucopyranose by the coupling constant (*J* = 8.0 Hz, Glc-1''') of the anomeric proton and GC analysis of hydrolysis followed trimethylsilylation of **4** in comparison with an authentic sample. The configurations of H-17 and the C-25 of **4** was assigned as the same as those of **3** by the NOE correlation of H-14/H-17 and the ∆_ab_ = 0.24 of the two geminal protons of H_2_-26. In the ^1^H-NMR spectrum, the proton signal at δ_H_ 4.91 (1H, t-like, *J* = 7.0 Hz, H-23), corresponded C-atom signal at δ_C_ 63.6 in the HSQC spectrum showed the presence of the hydroxyl at the C-23. On the basis of this evidence, the structure of **4** was revised as 26-*O*-β-d-glucopyranoside-3β,23,26-trihydroxy-(25*R*)-5α-furostan-20(22)-en-6-one. Besides the four new furostanol saponins, three known steroid saponins were isolated from this plant. Their structures were identified by compared the NMR properties with the reported data.

### 2.2. Cytotoxic Activity

The cytotoxicity of compounds **1**–**7** were tested against human cervical carcinoma cells (Hela) and human hepatocellular carcinoma cells (SMMC-7221) by the MTT method. The results revealed that compounds **2**–**6** were inactive (IC_50_ > 100 µM), while compounds **1** and **7** displayed cytotoxicity against Hela carcinoma cell lines with IC_50_ values of 18.79 ± 1.12 μM and 9.73 ± 1.64 μM, respectively and against SMMC-7221 cancer cell lines with IC_50_ values of 28.57 ± 1.57 μM and 21.54 ± 1.64 μM, respectively. Cisplatin used as the positive control had the IC_50_ values of 5.12 ± 0.26 μM (Hela) and 14.23 ± 0.44 μM (SMMC-7221). This investigation indicated that compound **7** which displays a spirostanol saponin structure showed stronger cytotoxic activity, whereas compound **1**, with a furostanol saponin structure, displayed moderate cytotoxic activity. Thus, according to our results, the spirostanol saponin skeleton exhibits higher cytotoxic activity than furostanol saponins, which is consistent with the literature [13].

## 3. Experimental Section

### 3.1. General

Optical rotations were measured with a Rudolph Research Analytical Autopol III automatic polarimeter (Rudolph Research Analytical, Hackettstown, NJ, USA) with MeOH as solvent. The IR spectra were conducted in KBr on a Bruker Tensor 27 spectrophotometer (Bruker, Billerica, MA, USA). The UV spectra were recorded on a Thermo Scientific Evolution 300 UV-visible spectrophotometer (Thermo Fisher Scientific, Waltham, MA, USA). The ^1^H- (500 MHz), ^13^C- (125 MHz) and 2D-NMR spectra were recorded on a Bruker DRX-500 instrument using TMS as internal standard. ESI-MS were collected on a Thermo Fisher LCQ Fleet mass spectrometer instrument (Thermo Fisher Scientific). HR-ESI-MS were carried out on a Bruker Bio-TOF-IIIQ mass spectrometer (Bruker). Absorbance value was measured by a Bio-Tek Epoch Absorbance Microplate Reader to calculate the inhibition rate (Bio-Tek, Winooski, VT, USA). 

Semi-preparative HPLC was run with a Waters 1525 pump and a Waters 2489 ultraviolet-visible detector using an Xterra^®^ prep MS C_18_ column (10 µm, 7.8 × 150 mm) (Waters, Milford, MA, USA). For column chromatography, silica gel (200–300 mesh; Qingdao Haiyang Chemical Co. Ltd, Qingdao, China), Rp-C_18_ (ODS-A, 50 µm, YMC, Yantai, China), and Sephadex LH-20 (GE Healthcare Bio-Science AB, Fairfield, CT, USA) were used. TLC (5 × 10 cm plates) were performed on GF254 (Qingdao Haiyang Chemical Co. Ltd) plates. Spots were detected after spraying with Ehrlich reagent (1 g 4-dimethylaminobenzaldehyde, in a mixture of 25 mL 37% hydrochloric acid and 75 mL methanol) followed by heating.

### 3.2. Plant Material

The rhizomes and roots of *S. scobinicaulis* were collected from Taibai Mountain of Shaanxi Province, China, in October 2010. The plant was identified by Zhenghai Wu of Northwest A&F University, Yangling, Shannxi, China. An authenticated voucher specimen (XB 00045) has been deposited at the Herbarium of Northwest A&F University, Yangling, Shannxi, China.

### 3.3. Extraction and Isolation

The air-dried powdered rhizomes and roots of *S. scobinicaulis* (62 kg) was extracted three times with 70% EtOH at room temperature for 48 h to obtain a concentrated extract, which was suspended in H_2_O and partitioned successively with PE, EtOAc and *n*-BuOH. The *n*-BuOH soluble extract (1792 g) was subjected to D101 macroporous adsorption resin and successively eluted with 30% EtOH, 50% EtOH and 70% EtOH to afford three fractions Frs.1–3. Fr.2 (600 g) was subjected to silica gel CC using a stepwise gradient of EtOAc–MeOH–H_2_O (from 15:1:0.5 to 3:1:0.5) to afford ten sub-fractions Frs. 2a–2j. Fr.2d (9.7 g) was chromatographed on silica gel CC eluting with EtOAc–MeOH (from 17:1 to 3:1) in gradient to yield four sub-fractions Frs.2d1–2d4 based on TLC. Fr.2d2 (1.2 g) was applied to Sephadex LH-20 CC with the eluent of MeOH to yield four sub-fractions Frs.2d2a–2d2d. Fr.2d2a (408 mg) was purified by RP-C_18_ CC and eluted with 65% MeOH–H_2_O to give compound **3** (52.7 mg). Fr.2d3 (1.6 g) was applied to Sephadex LH-20 CC with the eluent of MeOH to yield three sub-fractions Frs.2d3a–2d3c. Fr.2d3a (635 mg) was subjected to RP-C_18_ CC and eluted with 30%–70% MeOH–H_2_O to result four sub-fractions Frs.2d3a1–2d3a4. Fr.2d3a2 (156 mg) was purified by semi-preparative RP-C_18_ HPLC with the eluent of 48% MeOH–H_2_O (215 nm, flow rate: 3 mL/min) to give compound **4** (43.6 mg, t_R_ = 10.4 min). Fr.2d3a3 (238 mg) was purified by semi-preparative RP-C_18_ HPLC with the eluent 55% MeOH-H_2_O (215 nm, flow rate: 5 mL/min) to give compound **2** (55.8 mg, t_R_ = 7.9 min). Fr.2i (24.0 g) was chromatographed on Sephadex LH-20 CC with the eluent of MeOH to yield five sub-fractions Frs.2i1–2i5 based on TLC. Fr.2i1 (2.1 g) was applied to RP-C_18_ CC and eluted with 30%–70% MeOH–H_2_O to yield four sub-fractions Frs.2i1a–2i1d. Fr.2i1c (1.1 g) was chromatographed on silica gel CC eluting with EtOAc–MeOH–H_2_O (7:1:0.3–2:1:0.3) in gradient to yield four sub-fractions Frs.2i1c1–2i1c4. Fr.2i1c3 (223 mg) was purified by semi-preparative RP-C_18_ HPLC with the eluent of 53% MeOH–H_2_O (215 nm, flow rate: 3 mL/min) to give compound **5** (41.7 mg, t_R_ = 8.7 min) and **1** (42.3 mg, t_R_ = 9.3 min). Fr.2i1d (0.8 g) was chromatographed on silica gel CC eluting with EtOAc–MeOH–H_2_O (from 15:2:0.3 to 7:2:0.3) in gradient to give compound **7** (43.6 mg) and to yield five sub-fractions Frs.2i1d1-2i1d4. Fr.2i1d3 (339 mg) was purified by semi-preparative RP-C_18_ HPLC with the eluent of 50% MeOH–H_2_O (215 nm, flow rate: 2.5 mL/min) to give compound **6** (75.2 mg, t_R_ = 8.3 min).

### 3.4. New Compound Data

*26-O-*β*-*d*-Glucopyranoside-3*β,*26-dihydroxy-(25*R*)-5*α*-furostan-22-methoxyl-6-one-3-O-*α*-*l*-arabino-pyranosyl-(1****→****6)-*β*-*d*-glucopyranoside* (**1**). White amorphous powder, mp > 290 °C; ${\left[\alpha \right]}_{D}^{23.4}$ = −53.348° (c 0.115, CH_3_OH); IR υ_max_ (KBr): 3408, 2927, 1707 and 1044 cm^−1^; ^1^H-NMR and ^13^C-NMR data see Table 1; ESI-MS: *m/z* 941.4 [M+Na]^+^ and 917.3 [M−H]^−^; HR-ESI-MS: *m/z* 941.4727 [M+Na]^+^ (Calcd for C_45_H_74_NaO_19_: 941.4717).

**Table 1:** ^1^H- and ^13^C-NMR Data of Compounds **1**–**4**. (δ in ppm, *J* in Hz).

NO.	1		2		3		4	
	δ_H_^a^	δ_C_^b^	δ_H_^a^	δ_C_^b^	δ_H_^a^	δ_C_^b^	δ_H_^c^	δ_C_^d^
**1**	1.80 (m) 1.35 (m)	36.3	1.79 (m) 1.33 (m)	36.3	1.80 (m) 1.32 (m)	36.3	1.64 (m) 1.16 (m)	36.8
**2**	1.93 (m) 1.32 (m)	28.6	1.82 (m) 1.64 (m)	30.1	1.42 (m) 1.31 (m)	30.1	1.65 (m) 2.04 (m)	31.6
**3**	3.36 (m)	76.8	3.53 (m)	69.7	3.52 (m)	69.7	3.83 (m)	69.8
**4**	1.43 (m) 2.03 (m)	26.0	1.41 (m) 1.81 (m)	29.3	1.43 (m) 1.83 (m)	29.3	1.90 (m) 2.29 (m)	31.0
**5**	2.40 (d, 12.0)	56.0	2.38 (d, 11.0)	56.2	2.37 (dd, 12.5, 2.0)	56.2	2.26 (m)	56.8
**6**	–	212.0	–	212.0	–	212.0	–	209.7
**7**	2.16 (t, 13.0) 2.24 (m)	46.1	2.14 (t, 12.5) 2.25 (dd, 13.0, 4.5)	46.1	2.15 (m) 2.28 (dd, 13.0, 4.5)	46.2	2.02 (m) 2.35 (m)	46.8
**8**	2.00 (m)	37.4	2.00 (m)	37.4	1.97 (m)	37.3	1.81 (m)	37.0
**9**	1.40 (m)	53.4	1.40 (m)	53.4	1.36 (m)	53.4	1.15 (m)	53.5
**10**	–	40.8	–	40.6	–	40.6	–	40.7
**11**	1.42 (m) 1.70 (m)	21.0	1.42 (m) 1.68 (m)	21.0	1.42 m 1.72 m	21.2	1.52 (m) 1.73 (m)	21.5
**12**	1.28 (m) 1.82 (m)	39.1	1.25 (m) 1.82 (m)	39.2	1.37 m 1.88 m	39.0	1.22 (m)	39.2
**13**	–	41.1	–	41.1	–	43.6	–	43.9
**14**	1.43 (m)	56.0	1.41 (m)	56.0	1.45 (m)	54.6	1.06 (m)	54.6
**15**	1.30 (m) 1.95 (m)	31.1	1.30 (m) 1.94 (m)	31.2	1.45 (m) 2.15 (m)	33.5	1.38 (m) 1.97 (m)	33.9
**16**	4.40 (m)	80.8	4.41 (q–like, 7.5)	80.6	4.78 (m)	84.0	4.78 (m)	84.1
**17**	1.80 (m)	63.6	1.79 (m)	63.6	2.56 (d, 10.0)	64.0	2.48 (d, 10.5)	64.5
**18**	0.86 (s)	15.5	0.85 (s)	15.5	0.72 (s)	13.3	0.66 (s)	14.2
**19**	0.80 (s)	12.1	0.79 (s)	12.1	0.79 (s)	12.0	0.75 (s)	13.0
**20**	2.21 (m)	39.8	2.21 (m)	39.8	–	103.6	–	104.6
**21**	1.04 (d, 6.0)	14.7	1.03 (d, 7.0)	14.5	1.63 (s)	10.5	1.76 (s)	11.4
**22**	–	112.6	–	112.6	–	151.8		154.3
**23**	1.63 (m) 1.84 (m)	29.9	1.62 (m) 1.81 (m)	29.9	2.16 (m)	22.7	4.91 (t, 7.0)	63.6
**24**	1.16 (m) 1.62 (m)	27.6	1.16 (m) 1.61 (m)	27.2	1.27 (m) 1.65 (m)	30.6	1.74 (m) 2.38 (m)	39.5
**25**	1.76 (m)	33.6	1.75 (m)	33.6	1.78 (m)	32.7	2.44 (m)	30.7
**26**	3.40 (m) 3.76 (m)	74.6	3.40 (dd, 9.5, 6.5) 3.75 (dd, 9.5, 6.5)	74.6	3.41 (dd, 9.5, 6.0) 3.73 (dd, 9.5, 7.0)	74.4	3.79 (m) 4.03 (m)	75.3
**27**	0.98 (d, 5.0)	15.9	0.97 (d, 7.0)	16.0	0.97 (d, 6.5)	15.9	1.17 (d, 6.0)	17.6
OCH_3_	3.17 (s)	46.3	3.17 (s)	46.2	–	–	–	–
Glc–1'	4.42 (d, 6.5)	100.9	–	–	–	–	–	–
2'	3.19 (m)	73.7	–	–	–	–	–	–
3'	3.37 (m)	76.6	–	–	–	–	–	–
4'	3.30 (m)	70.4	–	–	–	–	–	–
5'	3.45 (m)	75.5	–	–	–	–	–	–
6'	3.83 (m) 4.09 (d, 11.5)	68.2	–	–	–	–	–	–
Ara–1''	4.34 (d, 6.0)	103.8	–	–	–	–	–	–
2''	3.60 (m)	71.0	–	–	–	–	–	–
3''	3.54 (m)	72.8	–	–	–	–	–	–
4''	3.71 (m)	68.0	–	–	–	–	–	–
5''	3.88 (m)	65.2	–	–	–	–	–	–
Glc–1'''	4.26 (d, 7.0)	103.2	4.26 (d, 8.0)	103.2	4.25 (d, 8.0)	103.1	4.85 (d, 8.0)	104.8
2'''	3.37 (m)	73.8	3.20 (t, 8.0)	73.8	3.21 (t, 8.5)	73.7	4.03 (m)	75.0
3'''	3.73 (m)	77.1	3.28 (m)	76.5	3.27 (m)	76.5	4.22 (m)	78.4
4'''	3.30 (m)	70.4	3.29 (m)	70.3	3.32 (m)	70.3	4.19 (m)	71.6
5'''	3.29 (m)	76.5	3.35 (m)	76.7	3.37 (m)	76.7	3.94 (m)	78.3
6'''	3.67 (m) 3.90 (m)	61.5	3.68 (dd, 12.0, 5.5) 3.88 (dd, 12.0, 1.5)	61.4	3.70 (dd, 12.0, 6.0) 3.88 (dd, 12.0, 2.0)	61.4	4.36 (m) 4.54 (d, 11.0)	62.7

^a^: Recorded at 500 MHz in CD_3_OD; ^b^: Recorded at 125 MHz in CD_3_OD; ^c^: Recorded at 500 MHz in Pyridine-*d*_5_; ^d^: Recorded at 100 MHz in Pyridine-*d*_5_.

*26-O-β-d-Glucopyranoside-3β,26-dihydroxy-(25R)-5α-furostan-22-methoxyl-6-one* (**2**)*.* White amorphous powder, mp > 290 °C; ${\left[\alpha \right]}_{D}^{23.8}$ = −40.029° (c 0.119, CH_3_OH); IR υ_max_ (KBr): 3423, 2927, 2872, 2853, 1713 and 1042 cm^−1^; ^1^H-NMR and ^13^C-NMR data see Table 1; ESI-MS: *m/z* 647.4 [M+Na]^+^ and 623.4 [M−H]^−^; HR-ESI-MS: *m/z* 647.3762 [M+Na]^+^ (Calcd for C_34_H_56_NaO_10_: 647.3766).

*26-O-β-d-Glucopyranoside-3β,26-dihydroxy-(25R)-5α-furostan-20(22)-en-6-one* (**3**)*.* White amorphous powder, mp > 290 °C; ${\left[\alpha \right]}_{D}^{24.1}$ = −6.152° (c 0.115, CH_3_OH); IR υ_max_ (KBr): 3421, 2943, 2915, 2865, 1697 and 1071 cm^−1^; ^1^H-NMR and ^13^C-NMR data see Table 1; ESI-MS: m/z 615.3 [M+Na]^+^, 591.3 [M−H]^−^ and 1183.5 [2M−H]^−^; HR-ESI-MS: *m/z* 615.3510 [M+Na]^+^ (Calcd for C_33_H_52_NaO_9_: 615.3504).

*26-O-β-d-Glucopyranoside-3β,23,26-trihydroxy-(23R,25R)-5α-furostan-20(22)-en-6-one* (**4**). White amorphous powder, mp > 290 °C; [α]_20.8*D*_ = −18.485° (c 0.13, CH_3_OH); IR υ_max_ (KBr) (cm^−1^): 3422, 2934, 2870, 1708, and 1077 cm^−1^; ^1^H-NMR and ^13^C-NMR data see Table 1; ESI-MS: *m/z* 631.55 [M+Na]^+^, 609.34 [M+H]^+^ and 1215.03 [2M−H]^−^; HR-ESI-MS: *m/z* 631.3450 [M+Na]^+^ (Calcd for C_33_H_52_NaO_10_: 631.3453).

*26-O-β-d-Glucopyranosyl-3β,22ξ,26-trihydroxy-(25R)-5α-furostan-6-one-3-O-α-l-arabinopyranosyl-(1→6)-β-d-glucopyranoside* (**5**). A white amorphous powder, molecular formula was C_44_H_72_O_19_. ESI-MS *m/z* 903.3 [M−1]^−^; ^1^H-NMR (CD_3_OD, 500 MHz) δ: 2.41 (1H, d, *J* = 12.0 Hz, H-5), 1.40 (1H, m, H-9), 1.45 (1H, m, H-14), 4.61 (1H, d, *J* = 7.5, H-16), 1.83 (1H, m, H-17), 0.85 (3H, s, H-18), 0.80 (3H, s, H-19), 1.04 (3H, d, *J* = 6.5 Hz, H-21), 3.41 (1H, m, H-26a), 3.75 (1H, m, H-26b), 0.97 (3H, d, *J* = 6.0 Hz, H-27), 4.42 (1H, d, *J* = 7.5 Hz, glc-H-1'), 4.10 (1H, d, *J* = 11.0 Hz, glc-H-6a'), 3.83 (1H, m, glc-H-6b'), 4.35 (1H, d, *J* = 6.5 Hz, glc-H-1''), 4.26 (1H, d, *J* = 7.5 Hz, glc-H-1'''); ^13^C-NMR (CD_3_OD, 125 MHz) δ: 36.3 (C-1), 28.6 (C-2), 76.7 (C-3), 26.0 (C-4), 56.0 (C-5), 212.0 (C-6), 46.1 (C-7), 37.4 (C-8), 53.4 (C-9), 40.8 (C-10), 21.0 (C-11), 39.2 (C-12), 41.1 (C-13), 56.0 (C-14), 31.2 (C-15), 80.6 (C-16), 62.6 (C-17), 15.5 (C-18), 12.1 (C-19), 39.4 (C-20), 14.4 (C-21), 110.5 (C-22), 35.6 (C-23), 27.2 (C-24), 33.5 (C-25), 74.6 (C-26), 16.0 (C-27), 100.9 (glc-C-1'), 73.7 (glc-C-2'), 76.6 (glc-C-3'), 70.4 (glc-C-4'), 75.5 (glc-C-5'), 68.2 (glc-C-6'), 103.8 (ara-C-1''), 71.0 (ara-C-2''), 72.8 (ara-C-3''), 68.0 (ara-C-4''), 65.2 (ara-C-5''), 103.2 (glc-C-1'''), 73.8 (glc-C-2'''), 77.1 (glc-C-3'''), 70.3 (glc-C-4'''), 76.5 (glc-C-5'''), 61.4 (glc-C-6''').

*26-O-β-d-Glucopyranosyl-3β,26-dihydroxy-(25R)-5α-furostan-20(22)-en-6-one-3-O-α-l-arabinopyranosyl-(1→6)-β-d-glucopyranoside* (**6**). A white amorphous powder, molecular formula was C_44_H_70_O_18_. ESI-MS *m/z* 885.3 [M−1]^−^; ^1^H-NMR (CD_3_OD, 500 MHz) δ: 2.41 (1H, d, *J* = 11.0 Hz, H-5), 2.27 (1H, dd, *J* = 13.0 and 4.5 Hz, H-7a), 2.19 (1H, m, H-7b), 1.41 (1H, m, H-9), 1.35 (1H, m, H-14), 4.77 (1H, m, H-16), 2.56 (1H, d, *J* = 10.0, H-17), 0.73 (3H, s, H-18), 0.80 (3H, s, H-19), 1.64 (3H, s, H-21), 3.38 (1H, m, H-26a), 3.72 (1H, m, H-26b), 0.98 (3H, d, *J* = 6.5 Hz, H-27), 4.43 (1H, d, *J* = 8.0 Hz, glc-H-1'), 4.10 (1H, d, *J* = 10.5 Hz, glc-H-6a'), 3.84 (1H, m, glc-H-6b'), 4.35 (1H, d, *J* = 6.5 Hz, glc-H-1''), 4.26 (1H, d, *J* = 8.0 Hz, glc-H-1'''); ^13^C-NMR (CD_3_OD, 125 MHz) δ: 36.3 (C-1), 28.6 (C-2), 76.7 (C-3), 26.0 (C-4), 56.0 (C-5), 212.0 (C-6), 46.3 (C-7), 37.3 (C-8), 53.4 (C-9), 40.8 (C-10), 21.2 (C-11), 39.0 (C-12), 43.7 (C-13), 54.6 (C-14), 33.5 (C-15), 84.0 (C-16), 64.0 (C-17), 13.3 (C-18), 12.1 (C-19), 103.6 (C-20), 10.5 (C-21), 151.8 (C-22), 22.7 (C-23), 30.6 (C-24), 32.7 (C-25), 74.4 (C-26), 16.0 (C-27), 100.9 (glc-C-1'), 73.7 (glc-C-2'), 76.5 (glc-C-3'), 70.3 (glc-C-4'), 75.4 (glc-C-5'), 68.2 (glc-C-6'), 103.8 (ara-C-1''), 71.0 (ara-C-2''), 72.8 (ara-C-3''), 68.1 (ara-C-4''), 65.3 (ara-C-5''), 103.1 (glc-C-1'''), 73.7 (glc-C-2'''), 77.1 (glc-C-3'''), 70.1 (glc-C-4'''), 76.5 (glc-C-5'''), 61.4 (glc-C-6''').

*Sieboldogenin-3-O-α-l-arabinopyranosyl-(1→6)-β-d-glucopyranoside* (**7**). A white amorphous powder, molecular formula was C_38_H_60_O_14_. ^1^H-NMR (DMSO, 500 MHz) δ: 2.33 (1H, d, *J* = 12.0 Hz, H-5), 1.30 (1H, m, H-9), 1.32 (1H, m, H-14), 4.31 (1H, q-like, *J* = 7.5, H-16), 0.73 (3H, s, H-18), 0.67 (3H, s, H-19), 0.92 (3H, d, *J* = 6.5 Hz, H-21), 3.41 (1H, m, H-26a), 3.51 (1H, m, H-26b), 3.73 (1H, d, *J* = 10.0 Hz, H-27a), 3.49 (1H, m, H-27b), 4.23 (1H, d, *J* = 7.5 Hz, glc-H-1'), 3.89 (1H, d, *J* = 11.0 Hz, glc-H-6a'), 3.49 (1H, m, glc-H-6b'), 4.21 (1H, d, *J* = 5.0 Hz, glc-H-1''); ^13^C-NMR (DMSO, 125 MHz) δ: 36.5 (C-1), 29.1 (C-2), 76.1 (C-3), 26.6 (C-4), 55.7 (C-5), 210.4 (C-6), 46.4 (C-7), 37.2 (C-8), 53.0 (C-9), 40.8 (C-10), 21.0 (C-11), 39.4 (C-12), 40.9 (C-13), 56.0 (C-14), 31.6 (C-15), 80.6 (C-16), 62.1 (C-17), 16.6 (C-18), 13.3 (C-19), 42.0 (C-20), 15.0 (C-21), 109.4 (C-22), 26.7 (C-23), 21.3 (C-24), 35.2 (C-25), 60.5 (C-26), 60.3 (C-27), 100.9 (glc-C-1'), 73.9 (glc-C-2'), 77.1 (glc-C-3'), 70.7 (glc-C-4'), 75.9 (glc-C-5'), 68.7 (glc-C-6'), 103.9 (ara-C-1''), 71.0 (ara-C-2''), 73.0 (ara-C-3''), 67.7 (ara-C-4''), 65.2 (ara-C-5'').

### 3.5. Acid Hydrolysis and Sugar Analysis

Compounds **1**–**4** (30 mg each) in 1 M HCl (MeOH–H_2_O, 1:1) were heated under reflux for 8 h. After removal of the solvent, the residue was partitioned between CHCl_3_ and H_2_O. The water layer was neutralized with 5% NaOH and desalted (Sephadex LH-20, MeOH). The desalted segment was found to contain d-glucose and l-arabinose by TCL comparison with authentic sample (EtOAc–MeOH–H_2_O–AcOH = 6.5:2.0:1.5:1.5) and by measurement of their optical rotation ([α]_20 *D*_ + 60°, c = 0.15, H_2_O; ${\left[\alpha \right]}_{D}^{20}$ = +118°, c = 0.15, H_2_O) for compound **1**, and was only found to contain d-glucose for compounds **2**–**4**.

### 3.6. GC Analysis of the Sugar Moieties in **1**–**4**

Compounds **1**–**4** (each 4 mg) in 1 M HCl (5 mL, dioxane–H_2_O 1:1, v/v) were heated at 95 °C for 6 h, respectively. The reaction mixtures were evaporated *in vacuo*. The residues were dissolved in water and then extracted with CHCl_3_ for three times, respectively. After evaporating the aqueous phases to dryness *in vacuo*, the residues were dissolved in pyridine (5 mL) and 1-(trimethylsilyl)-imidazole (0.5 mL) at room temperature for 30 min. The reaction mixtures were dried with a stream of N_2_. The residues were partitioned between CHCl_3_ and H_2_O. The organic layers were subjected to GC analysis using an l-Chirasil-Val column (0.32 mm × 25 m) [14]. Temperature of the injector and detector was 200 °C. A temperature gradient system was used for the oven; the initial was temperature was maintained at 100 °C for 1 min and then increasing up to 180 °C at a rate of 5 °C/min. Peaks of the hydrolysate of **1** were detected at 8.92 and 9.80 min (l-arabinose) and 14.71 min (d-glucose). However, respectively detecting only one peak at 14.73, 14.72 and 14.72 min in the hydrolysate of **2**, **3** and **4**, suggested that all the sugar moieties of **2**, **3** and **4** are d-glucose. Retention times for authentic samples of d-glucose and l-arabinose (Sigma Aldrich, St. Louis, MO, USA) after being treated in the same manner with 1-(trimethylsilyl)-imidazole in pyridine were detected at 8.90 and 9.78 min (l-arabinose), 14.71 min (d-glucose).

### 3.7. Cytotoxicity Assay

The cytotoxic activities of compounds **1**–**7** were performed against human cervical carcinoma cells (Hela) and human hepatocellular carcinoma cells (SMMC-7221) by MTT [3-(4,5-dimethylthiazol-2-yl)-2,5-diphenyltetrazolium bromide] assay [15], with cisplatin as positive control. Cell lines were maintained in Dulbecco’s Modified Eagle Medium (DMEM) medium containing 10% fetal bovine serum and were cultivated in humidified incubator at 5% CO_2_ and 37 °C. The human cancer cells in the log phase of their growth cycle (2.5 × 10^4^ cells/mL) were added to each well of the 96-well plates (100 µL/well) and were cultivated for 16 h. The test compounds were then added and the cells were further incubated for 48 h. After, 20 µL of MTT solution (5 mg/mL in PBS) were added to each well and the cells were incubated for addtitional 4 h. The supernatant was discarded and after washing with PBS, DMSO (100 µL/well) was added to dissolve the formazan crystals. The optical density (OD) was measured by enzyme immunoassay instrument at 570 nm. Each assay was done in triplicate.

## 4. Conclusions

Four new furostanol saponins **1**–**4** were isolated and identified from the rhizomes and roots of *Smilax scobinicaulis*. Besides, two known furostanol saponins **5**–**6** and one known spirostanol saponin **7** are described for the first time in this plant. Among the isolated saponins, compounds **1** and **7** displayed cytotoxicity against the Hela and SMMC-7221 cancer cell lines. The discovery of these new bioactive compounds further expands our knowledge of the structural diversity of the bioactive products produced by the plant *Smilax scobinicaulis*, and lays a chemical foundation for its pharmacological application.

## Supplementary Materials

Supplementary materials can be accessed at: http://www.mdpi.com/1420-3049/19/12/20975/s1.
