# Supplementary material for: Four New Furostanol Saponins from the Rhizomes and Roots of Smilax scobinicaulis and Their Cytotoxicity
Source: Molecules. 2014 Dec 15;19(12):20975–87. doi: 10.3390/molecules191220975 (PMC6271347; doi:10.3390/molecules191220975)

Supplementary Materials

Figure S1. ^{1}H-NMR spectrum of compound **1**.

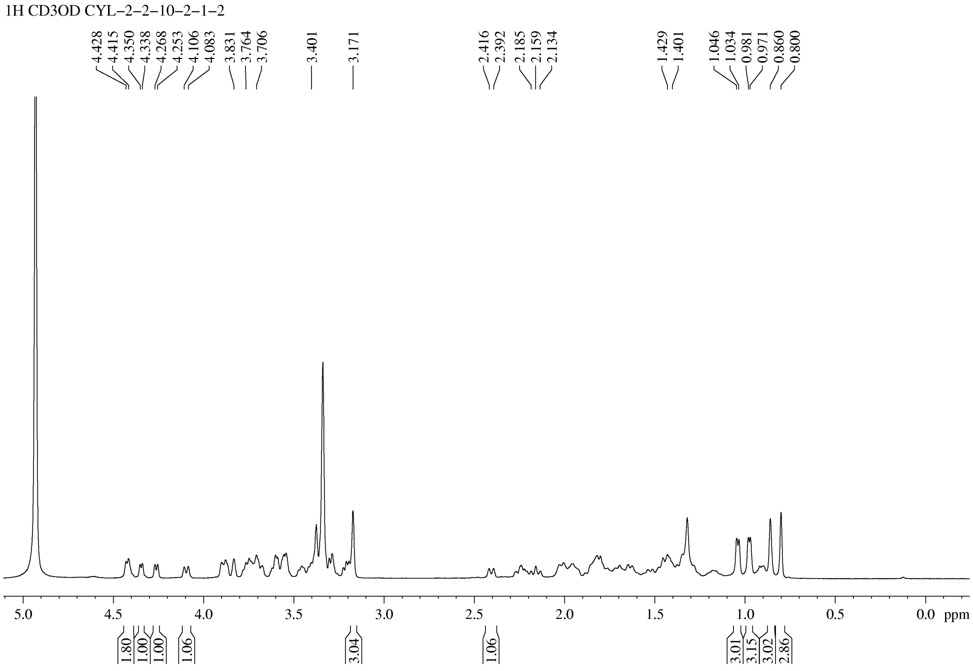

Figure S2. ^{13}C-NMR spectrum of compound **1**.

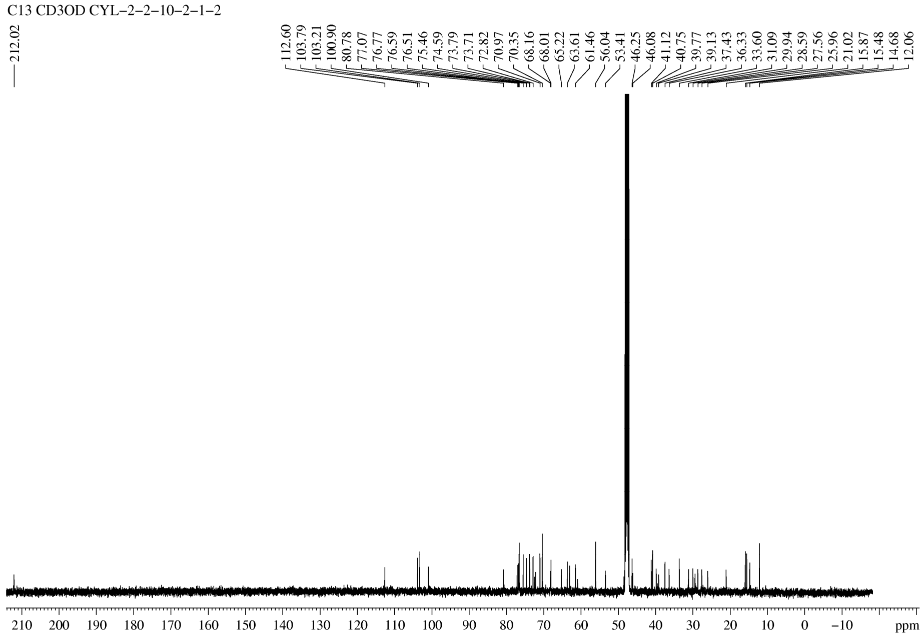

Figure S3. DEPT spectrum of compound **1**.

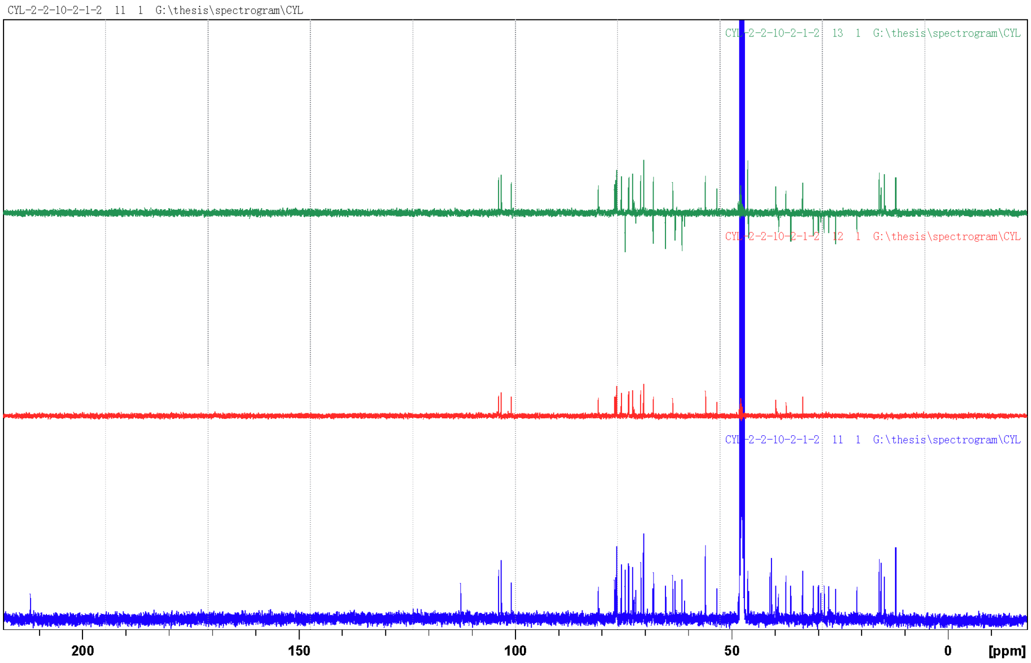

Figure S4. COSY spectrum of compound **1**.

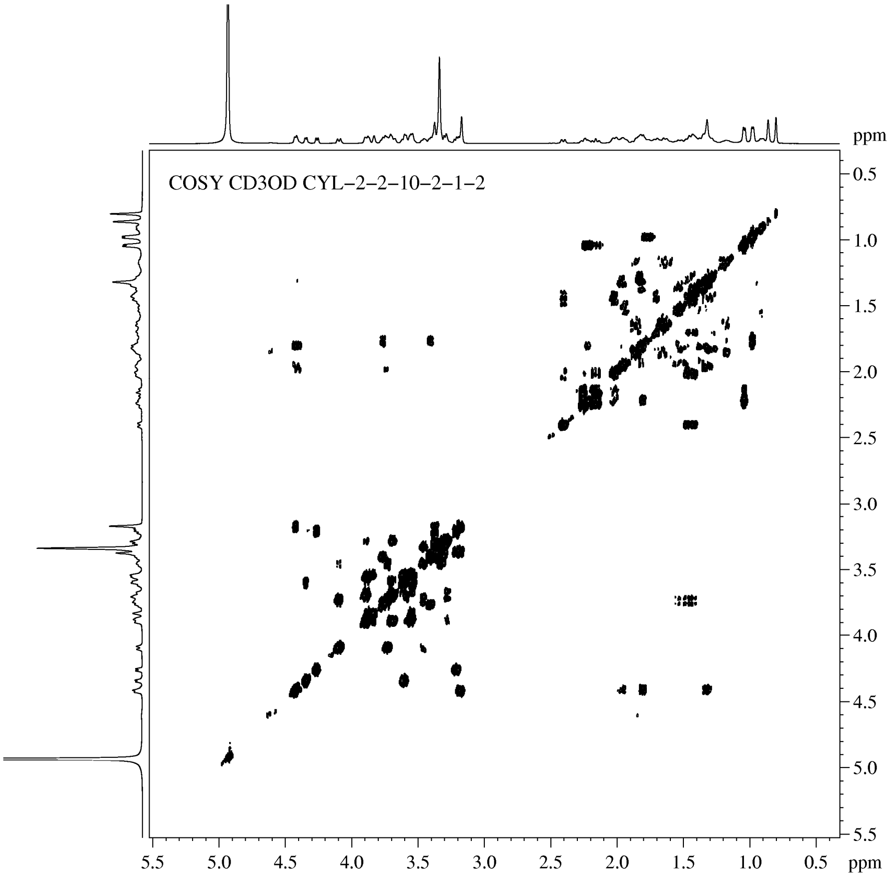

Figure S5. HSQC spectrum of compound **1**.

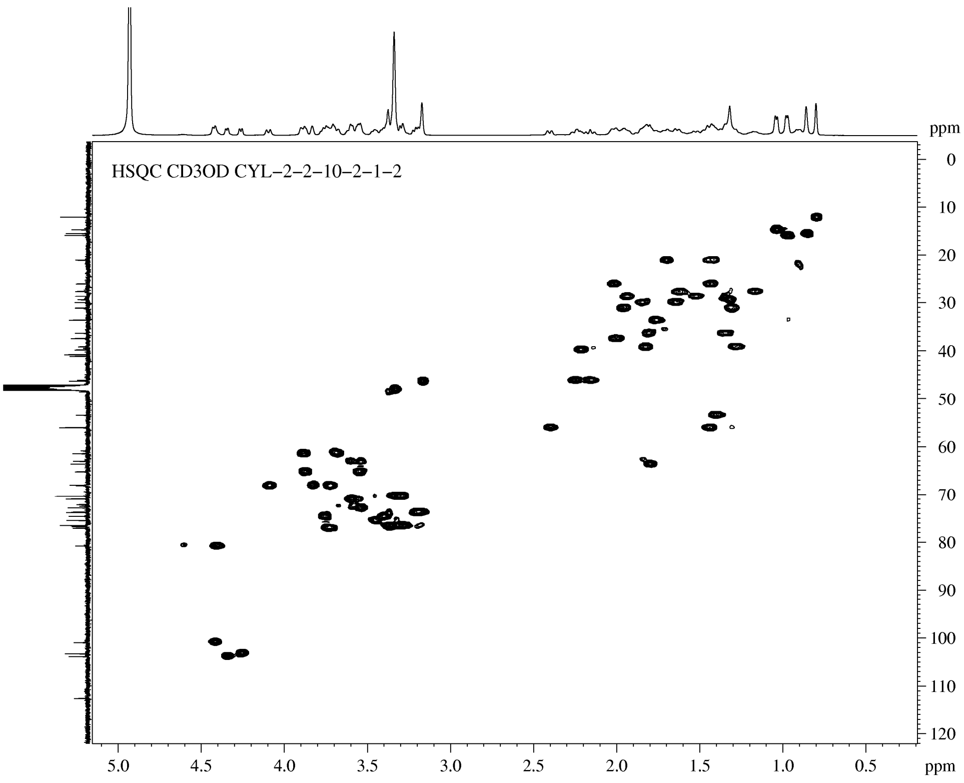

Figure S6. HMBC spectrum of compound **1**.

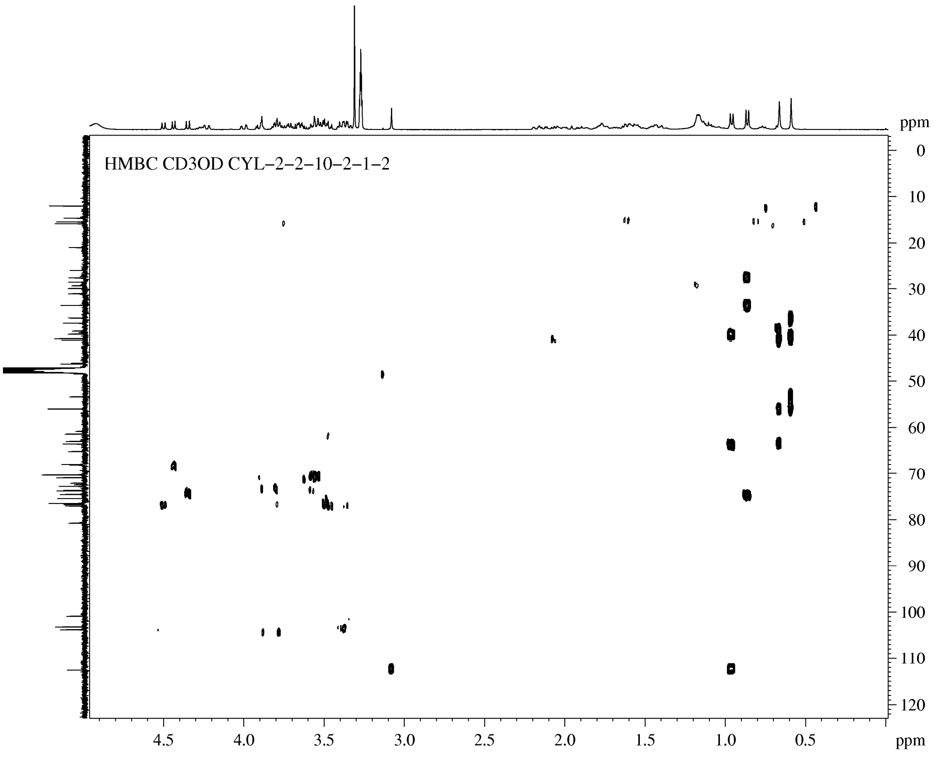

Figure S7. NOESY spectrum of compound **1**.

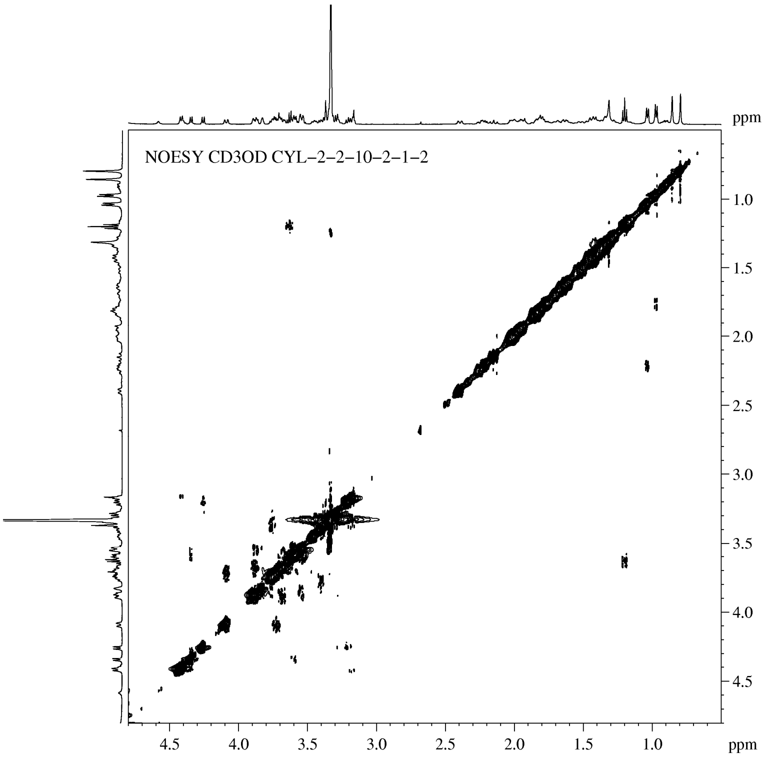

Figure S8. ESI-MS spectrum of compound **1**.

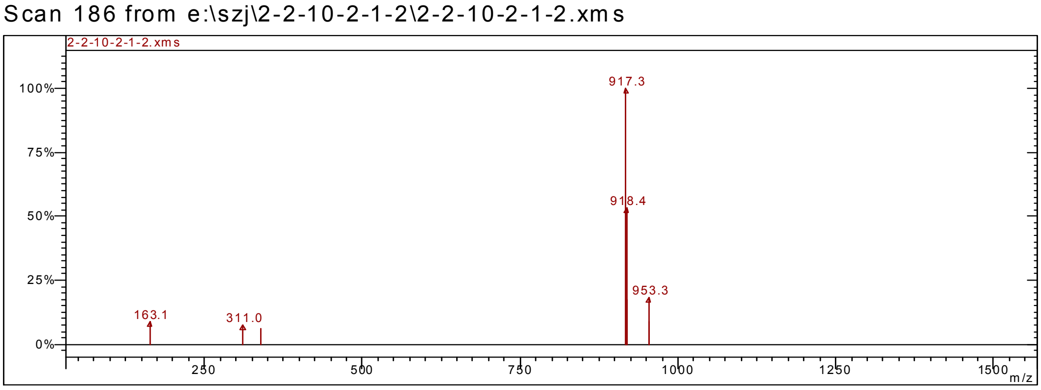

Spectrum from e:\szj\2-2-10-2-1-2\2-2-10-2-1-2.xms
Scan No: 186, Time: 3.347 minutes
3 points averaged. Background corrected.
Comment: 3.347 min. Scans: 183-187 100.0:1500.0>(-) RIC: 1.135e+8
Pair Count: 8 MW: 0 Formula: None
CAS No: None Acquired Range: 100.0 - 1500.0 m/z

Method Description: ESI
Scan 1 Channel Description: 100.0:1500.0> ESI;CID 175.0;Det 1100;WIQ1
Scan 2 Channel Description: 100.0:1500.0>(-) ESI;CID -165.0;Det 1100;W
Scan Information: cp = 0.1 mTorr
Precursor Mass Range: 100.0 - 1500.0 m/z

Ion	Int	Norm	Ion	Int	Norm	Ion	Int	Norm
163.1	1.317e+6	90	917.3	1.460e+7	999	953.3	2.695e+6	184
311.0	1.118e+6	77	918.4	7.761e+6	531	954.6	1.265e+6	87
339.3	904857	62	919.5	2.523e+6	173			

(−) ESI

Figure S8. *Cont.*

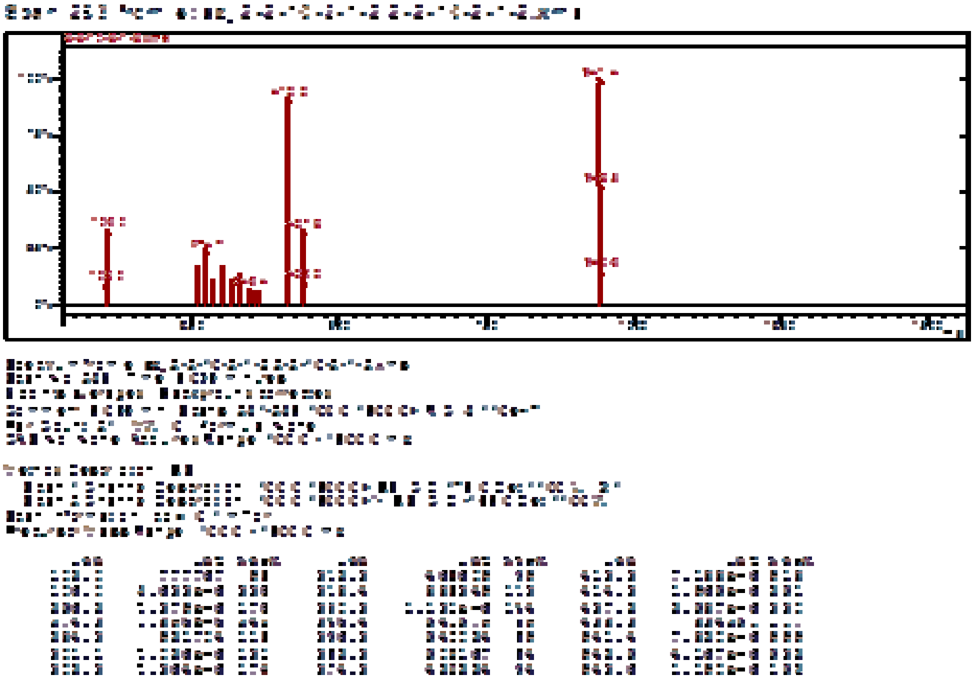

(+) ESI

Figure S9. HR-ESI-MS spectrum of compound **1**.

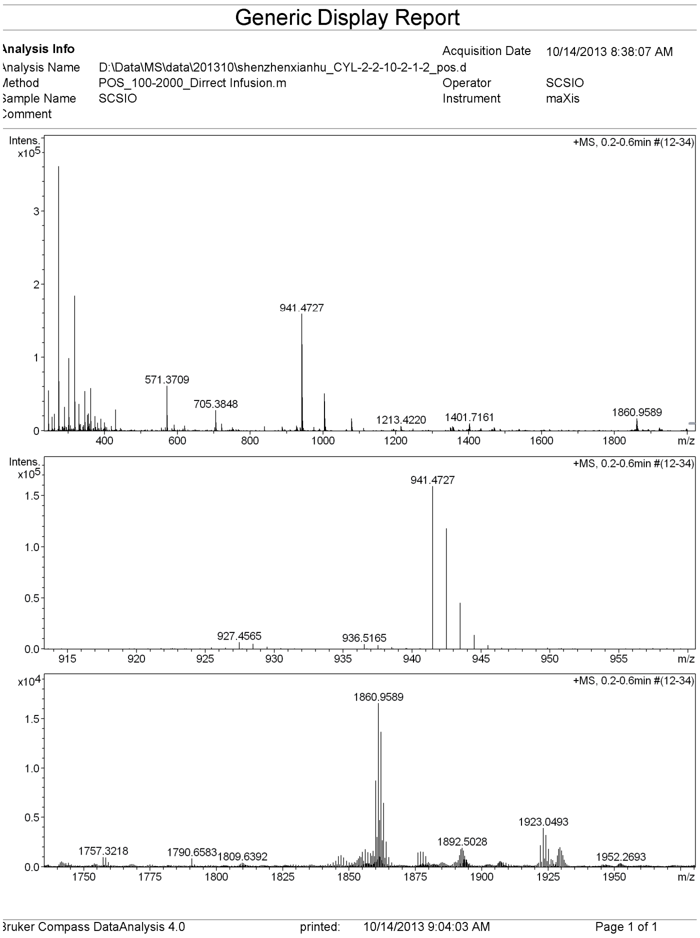

Figure S9. *Cont.*

Mass Spectrum SmartFormula Report

Analysis Info

		Acquisition Date	10/14/2013 8:38:07 AM
Analysis Name	D:\Data\MS\data\201310\shenzhenxianhu_CYL-2-2-10-2-1-2_pos.d		
Method	POS_100-2000_Dirrect Infusion.m	Operator	SCSIO
Sample Name	SCSIO	Instrument / Ser#	maXis 29
Comment			

Acquisition Parameter

Source Type	ESI	Ion Polarity	Positive	Set Nebulizer	0.3 Bar
Focus	Not active	Set Capillary	4500 V	Set Dry Heater	180 °C
Scan Begin	100 m/z	Set End Plate Offset	-500 V	Set Dry Gas	4.0 l/min
Scan End	2000 m/z	Set Collision Cell RF	2000.0 Vpp	Set Divert Valve	Waste

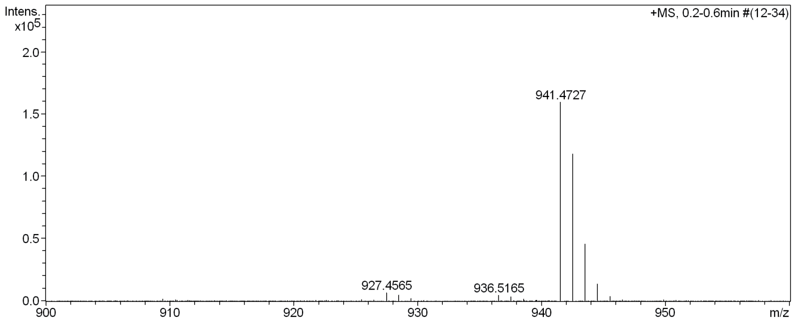

Meas. m/z	#	Formula	Score	m/z	err [mDa]	err [ppm]	mSigma	rdb	e⁻ Conf	N-Rule
919.4914	1	C 45 H 75 O 19	100.00	919.4897	-1.7	-1.8	567.3	8.5	even	ok
941.4727	1	C 45 H 74 Na O 19	100.00	941.4717	-1.0	-1.1	121.5	8.5	even	ok

Bruker Compass DataAnalysis 4.0 printed: 10/14/2013 9:02:58 AM Page 1 of 1

Figure S10. IR spectrum of compound **1**.

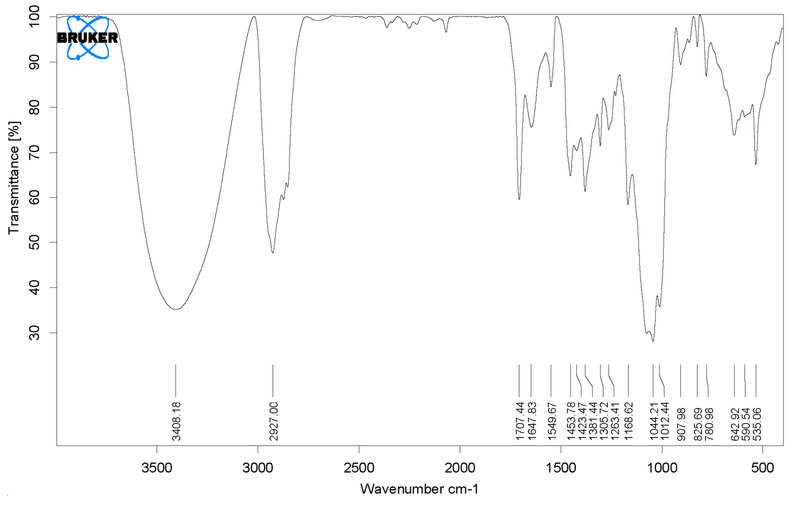

E:\DATABASE\MEAS\ \2210212.0 2210212 Instrument type and / or accessory

Page 1/1

Figure S11. ^{1}H-NMR spectrum of compound **2**.

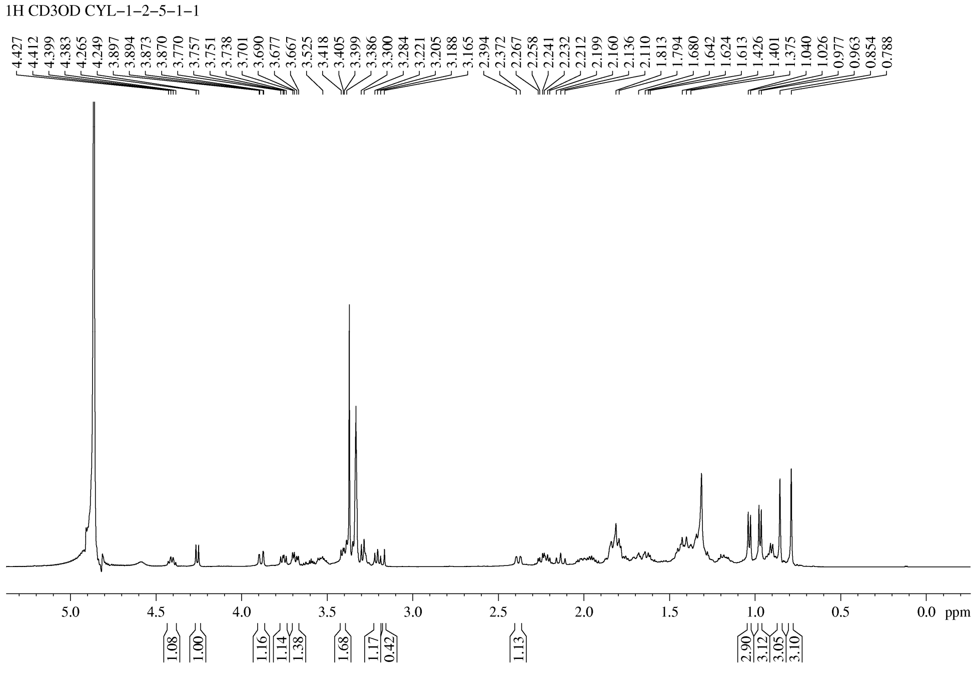

Figure S12. ^{13}C-NMR spectrum of compound **2**.

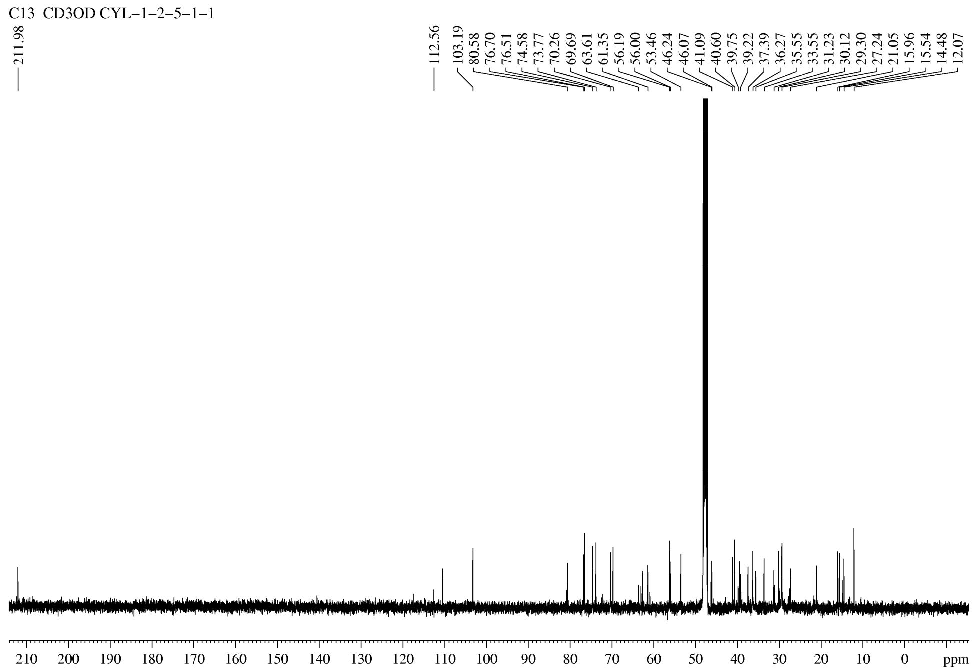

Figure S13. DEPT spectrum of compound **2**.

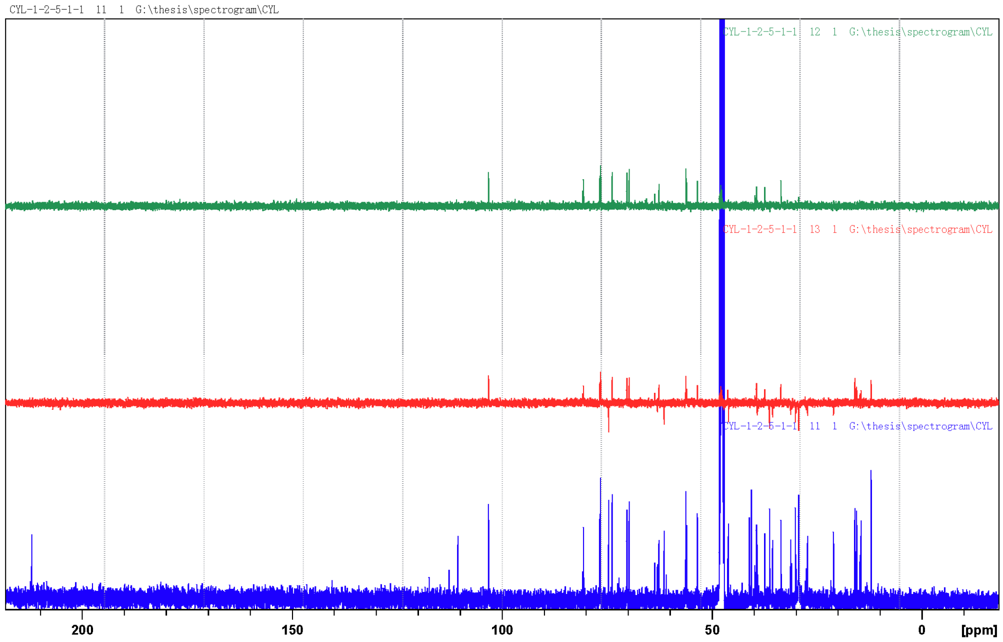

Figure S14. COSY spectrum of compound **2**.

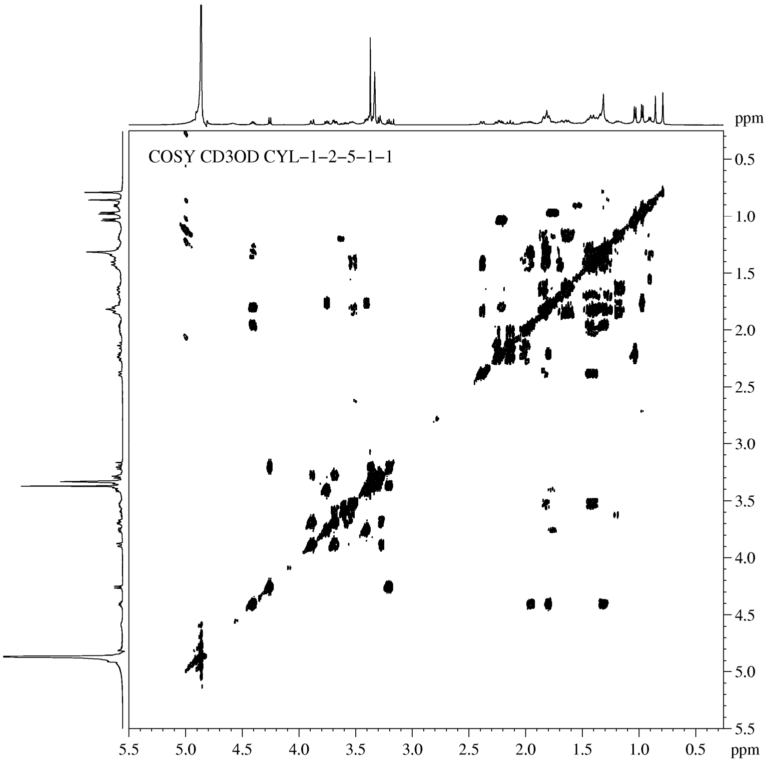

Figure S15. HSQC spectrum of compound **2**.

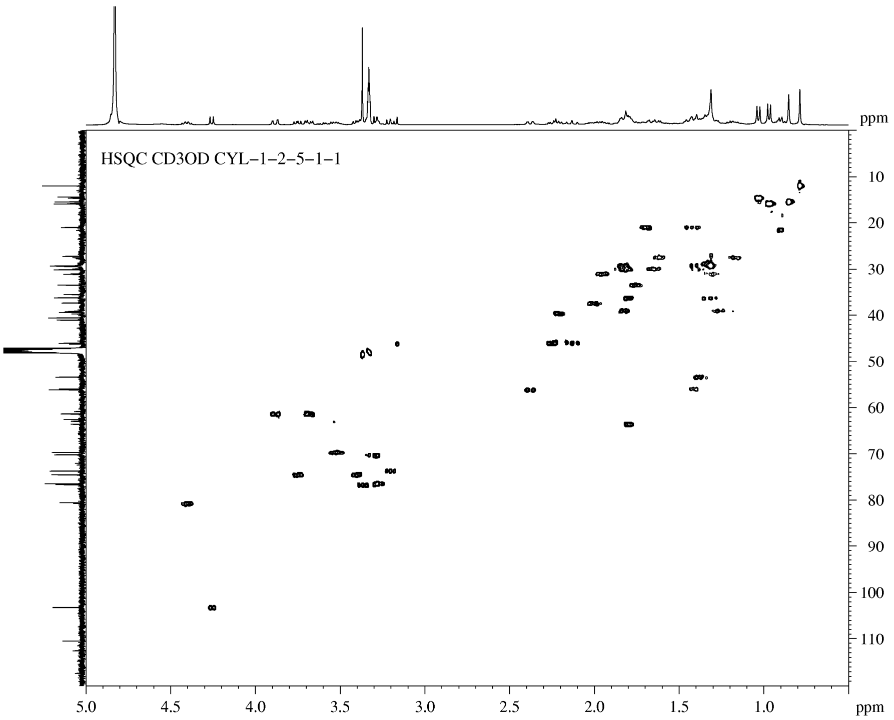

Figure S16. HMBC spectrum of compound **2**.

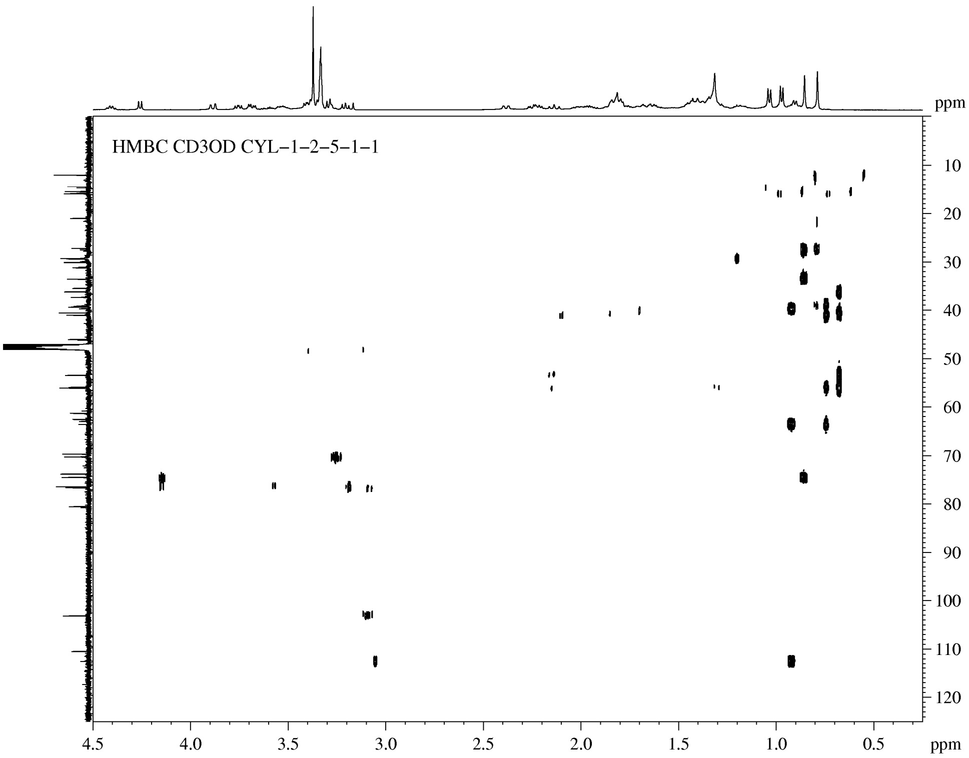

Figure S17. NOESY spectrum of compound **2**.

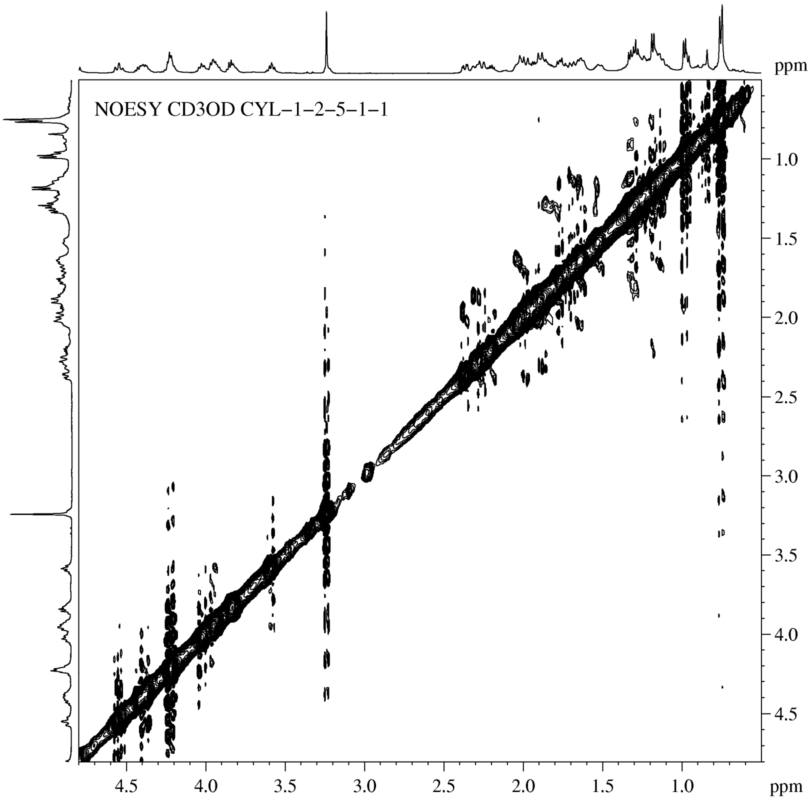

Figure S18. ESI-MS spectrum of compound **2**.

Scan 267 from e:\szj\1-2-5-1-1\1-2-5-1-1.xms

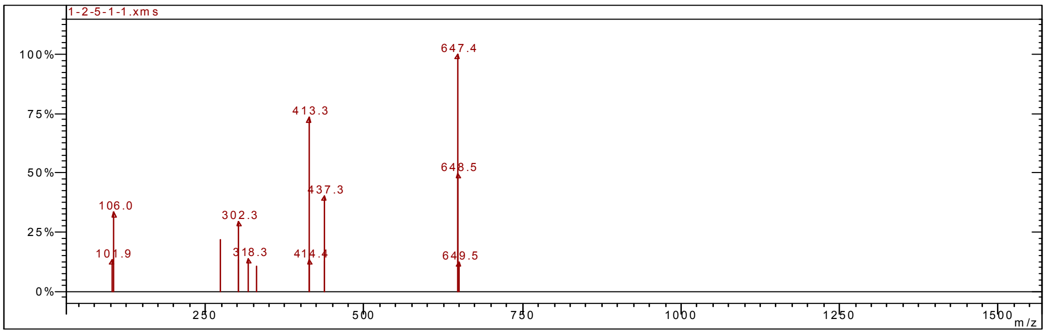

Spectrum from e:\szj\1-2-5-1-1\1-2-5-1-1.xms
Scan No: 267, Time: 4.808 minutes
3 points averaged. Background corrected.
Comment: 4.808 min. Scans: 265-269 100.0:1500.0> RIC: 1.411e+8
Pair Count: 14 MW: 0 Formula: None
CAS No: None Acquired Range: 100.0 - 1500.0 m/z

Method Description: ESI
Scan 1 Channel Description: 100.0:1500.0> ESI;CID 175.0;Det 1150;WIQ1
Scan 2 Channel Description: 100.0:1500.0>(-) ESI;CID -140.0;Det 1150;W
Scan Information: cp = 0.1 mTorr
Precursor Mass Range: 100.0 - 1500.0 m/z

Ion	Int	Norm	Ion	Int	Norm	Ion	Int	Norm
101.9	1.514e+6	133	318.3	1.580e+6	139	438.4	1.450e+6	127
106.0	3.794e+6	333	330.4	1.191e+6	105	647.4	1.138e+7	999
274.3	2.498e+6	219	413.3	8.340e+6	732	648.5	5.645e+6	496
301.2	1.524e+6	134	414.4	1.506e+6	132	649.5	1.401e+6	123
302.3	3.353e+6	294	437.3	4.605e+6	404			

(+) ESI

Figure S18. *Cont.*

Scan 146 from e:\szj\1-2-5-1-1\1-2-5-1-1.xms

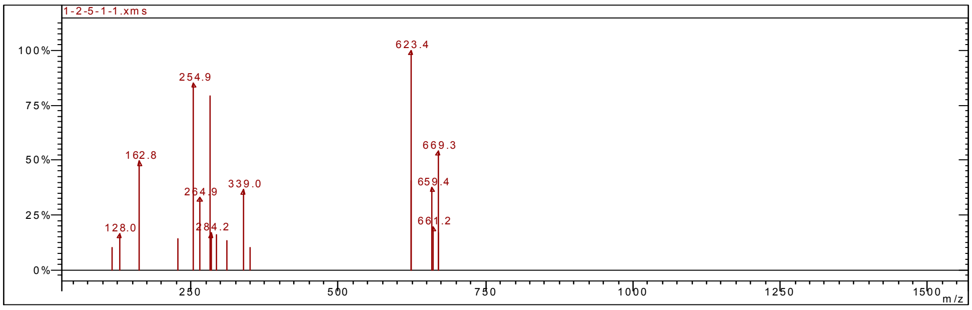

Spectrum from e:\szj\1-2-5-1-1\1-2-5-1-1.xms
Scan No: 146, Time: 2.613 minutes
3 points averaged. Background corrected.
Comment: 2.613 min. Scans: 143-147 100.0:1500.0>(-) RIC: 2.227e+8
Pair Count: 18 MW: 0 Formula: None
CAS No: None Acquired Range: 100.0 - 1500.0 m/z

Method Description: ESI
Scan 1 Channel Description: 100.0:1500.0> ESI;CID 175.0;Det 1150;WIQ1
Scan 2 Channel Description: 100.0:1500.0>(-) ESI;CID -140.0;Det 1150;W
Scan Information: cp = 0.1 mTorr
Precursor Mass Range: 100.0 - 1500.0 m/z

Ion	Int	Norm	Ion	Int	Norm	Ion	Int	Norm
115.0	1.058e+6	104	283.1	8.052e+6	792	623.4	1.016e+7	999
128.0	1.676e+6	165	284.2	1.703e+6	167	624.4	4.158e+6	409
162.8	5.032e+6	495	292.8	1.648e+6	162	659.4	3.819e+6	375
226.8	1.425e+6	140	311.1	1.348e+6	133	661.2	2.008e+6	197
254.9	8.642e+6	850	339.0	3.706e+6	364	669.3	5.506e+6	541
264.9	3.382e+6	333	351.2	1.024e+6	101	670.4	1.711e+6	168

(−) ESI

Figure S19. HR-ESI-MS spectrum of compound **2**.

Generic Display Report

Analysis Info

Analysis Name	D:\Data\MS\data\201310\shenzhenxianhu_CYL-1-2-5-1-1_pos.d	Acquisition Date	10/14/2013 8:31:04 AM
Method	POS_100-2000_Dirrect Infusion.m	Operator	SCSIO
Sample Name	SCSIO	Instrument	maXis
Comment			

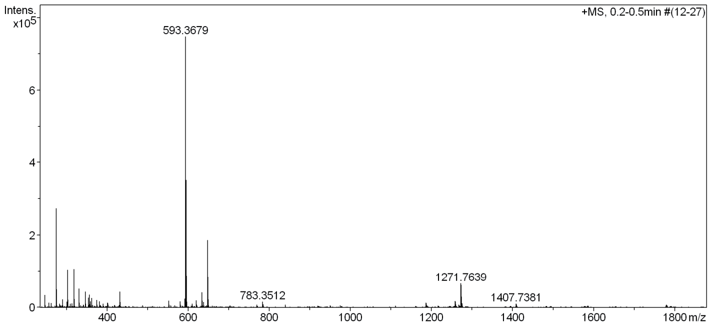

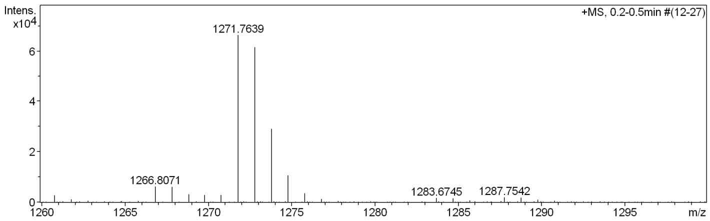

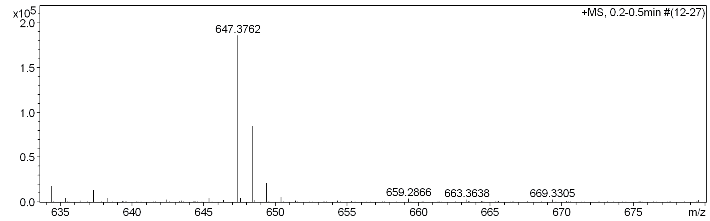

Figure S19. *Cont.*

Mass Spectrum SmartFormula Report

Analysis Info

Analysis Name	D:\Data\MS\data\201310\shenzhenxianhu_CYL-1-2-5-1-1_pos.d	Acquisition Date	10/14/2013 8:31:04 AM
Method	POS_100-2000_Dirrect Infusion.m	Operator	SCSIO
Sample Name	SCSIO	Instrument / Ser#	maXis 29
Comment			

Acquisition Parameter

Source Type	ESI	Ion Polarity	Positive	Set Nebulizer	0.3 Bar
Focus	Not active	Set Capillary	4500 V	Set Dry Heater	180 °C
Scan Begin	100 m/z	Set End Plate Offset	-500 V	Set Dry Gas	4.0 l/min
Scan End	2000 m/z	Set Collision Cell RF	2000.0 Vpp	Set Divert Valve	Waste

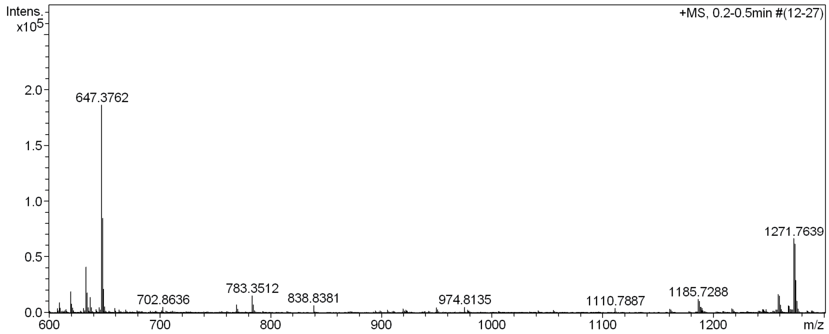

Meas. m/z	#	Formula	Score	m/z	err [mDa]	err [ppm]	mSigma	rdb	e⁻ Conf	N-Rule
647.3762	1	C 34 H 56 Na O 10	100.00	647.3766	0.4	0.6	41.2	6.5	even	ok
1271.7639	1	C 68 H 112 Na O 20	100.00	1271.7639	0.0	0.0	96.3	12.5	even	ok

Figure S20. IR spectrum of compound **2**.

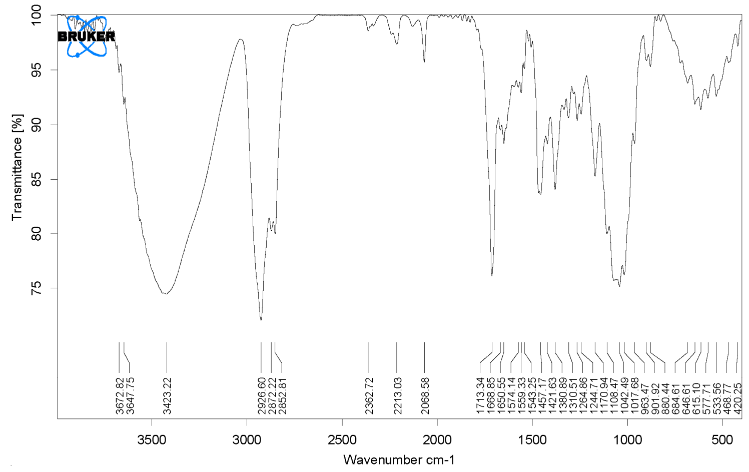

E:\DATABASE\MEAS\ \12511.0 12511 Instrument type and / or accessory

Page 1/1

Figure S21. ^{1}H-NMR spectrum of compound **3**.

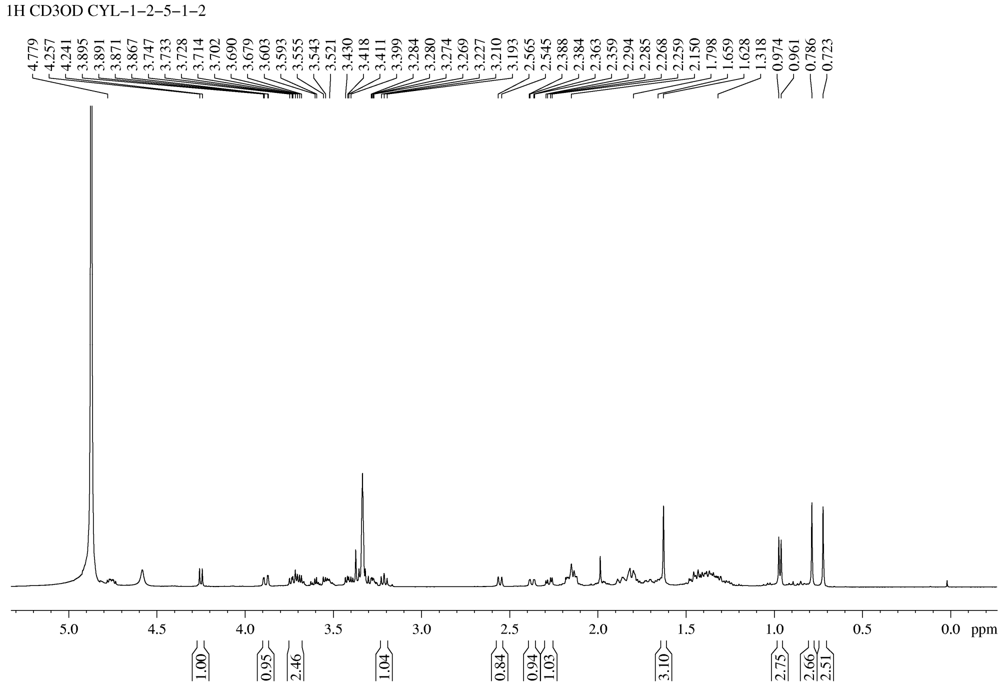

Figure S22. ^{13}C-NMR spectrum of compound **3**.

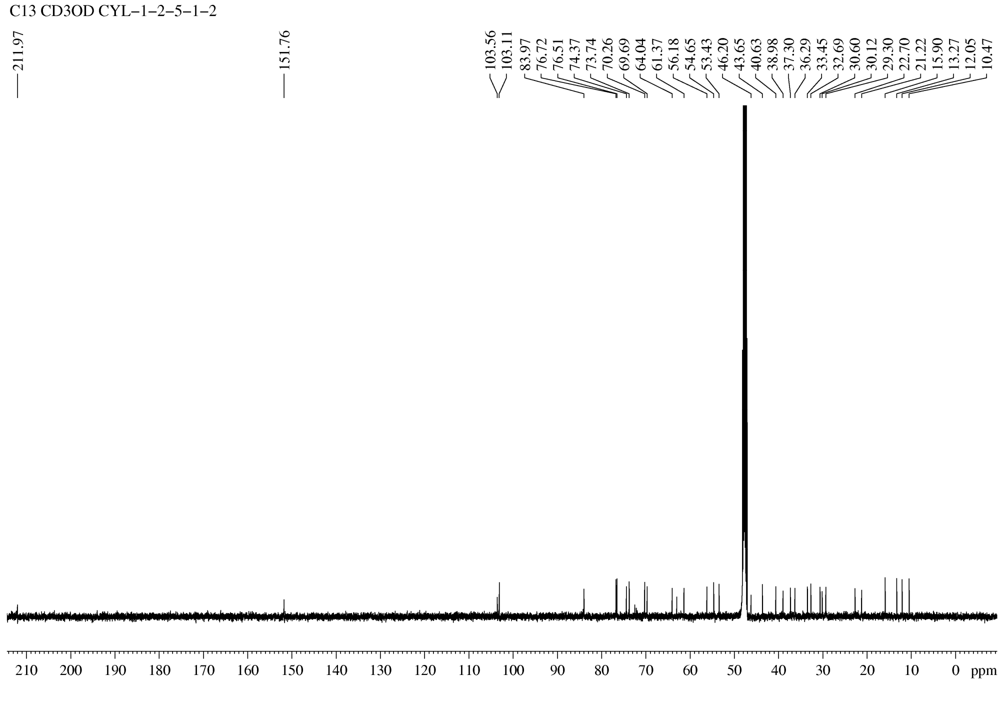

Figure S23. DEPT spectrum of compound **3**.

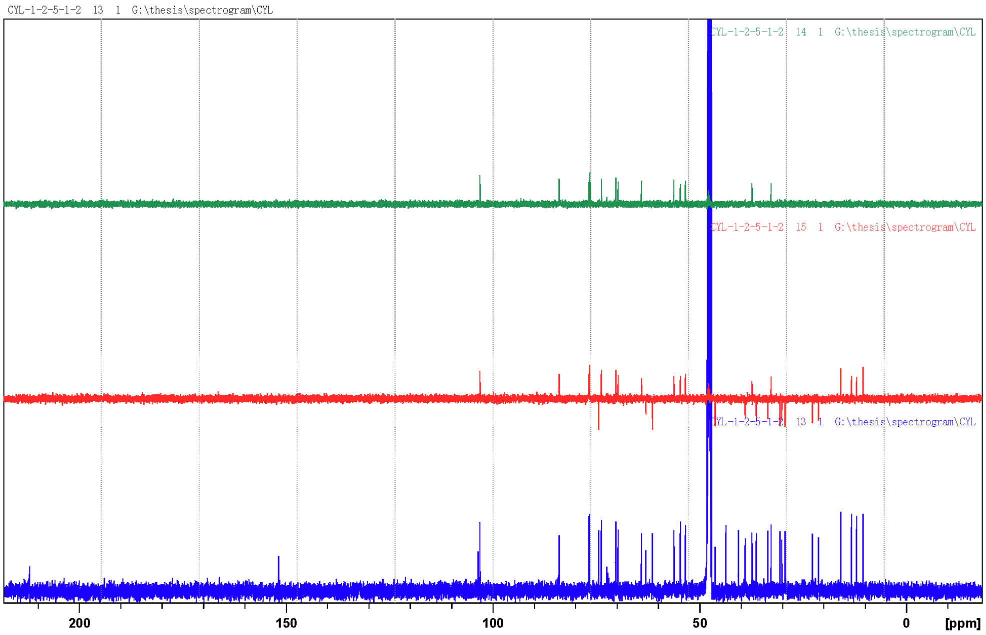

Figure S24. COSY spectrum of compound **3**.

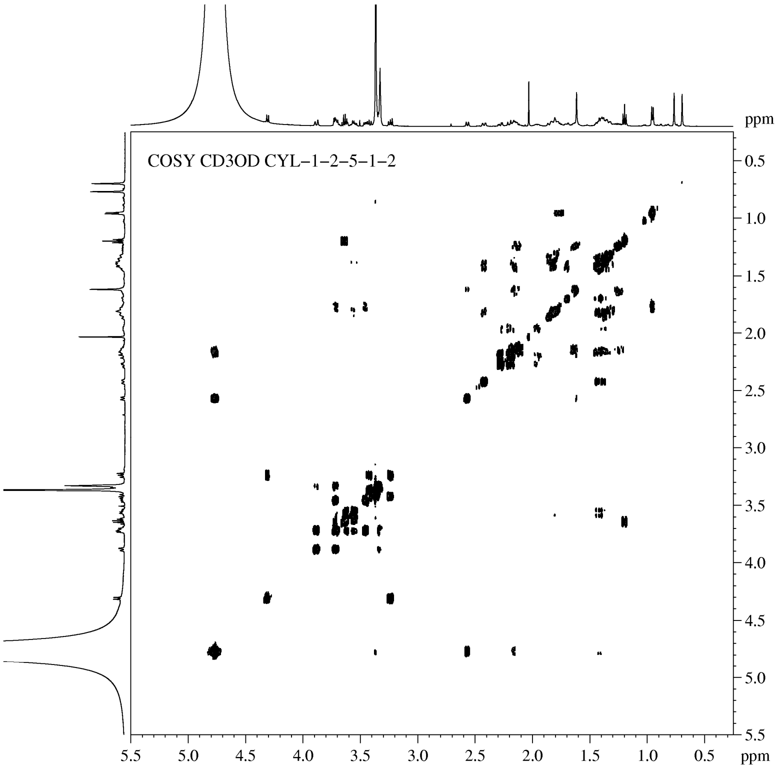

Figure S25. HSQC spectrum of compound **3**.

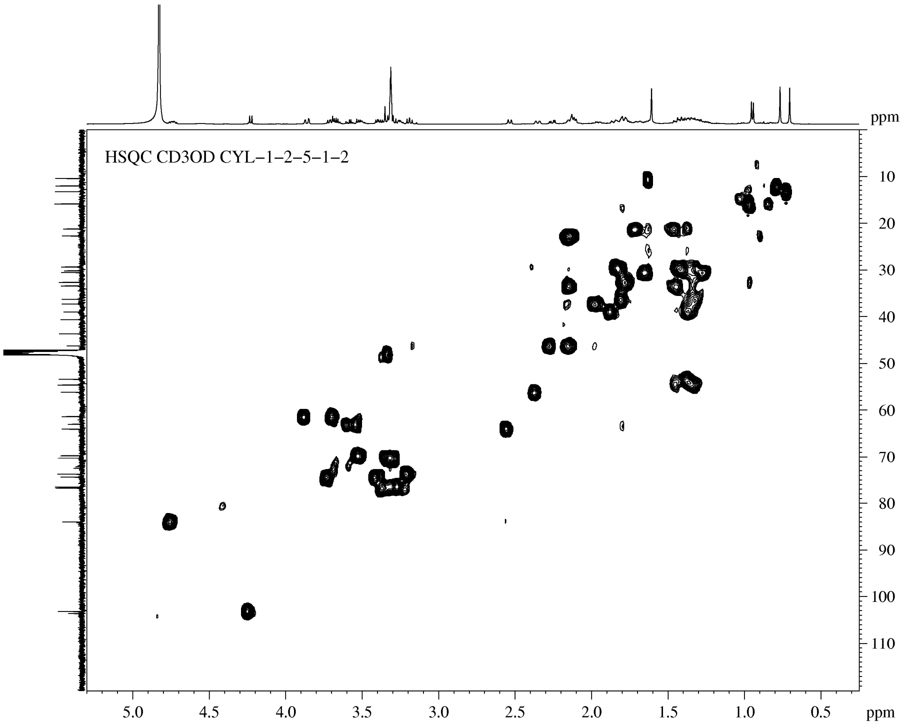

Figure S26. HMBC spectrum of compound **3**.

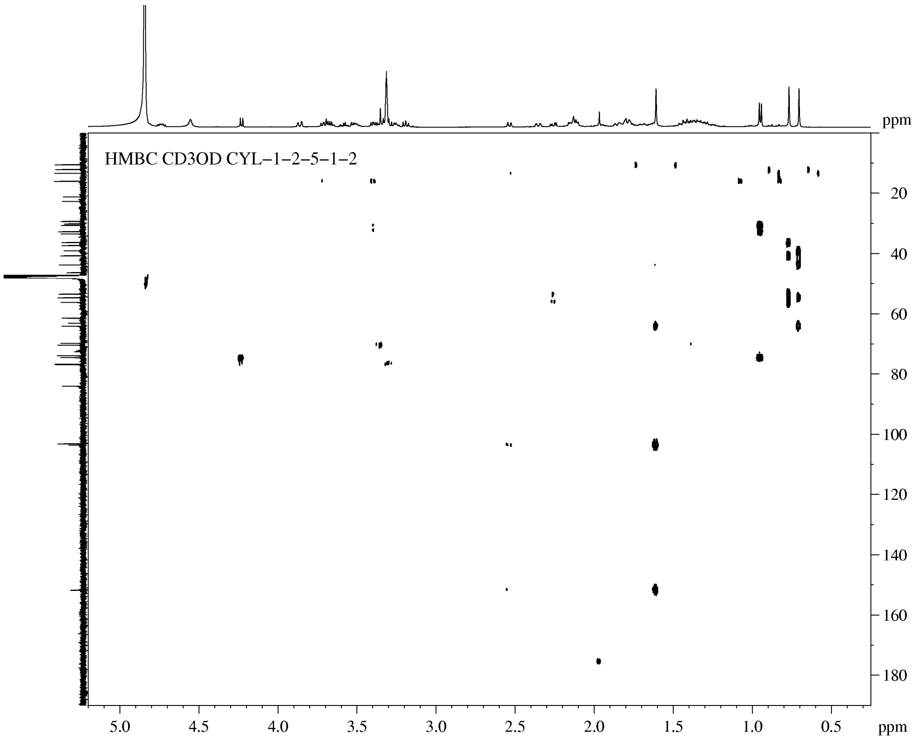

Figure S27. NOESY spectrum of compound **3**.

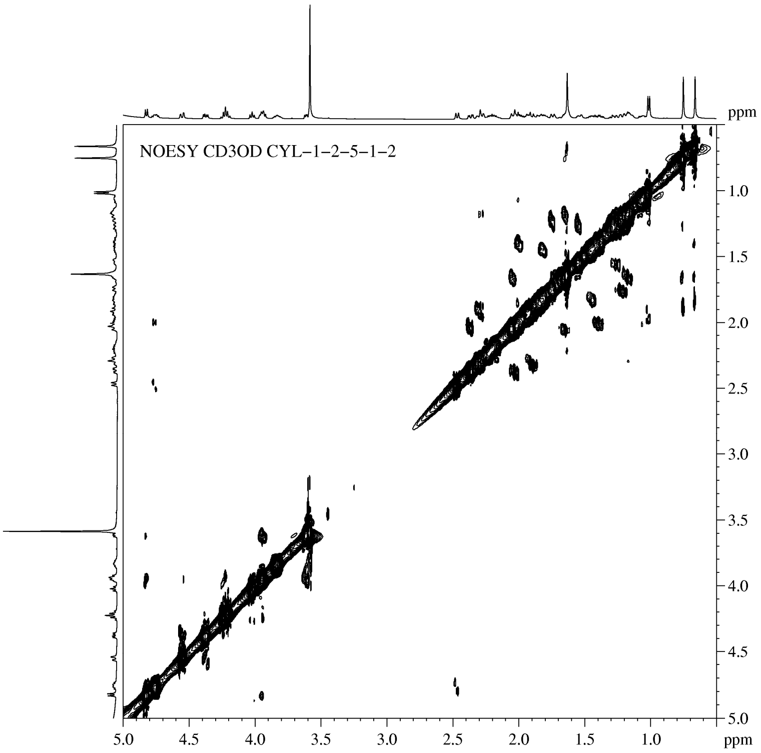

Figure S28. ESI-MS spectrum of compound **3**.

Scan 301 from e:\szj\1-2-5-1-2\1-2-5-1-2.xms

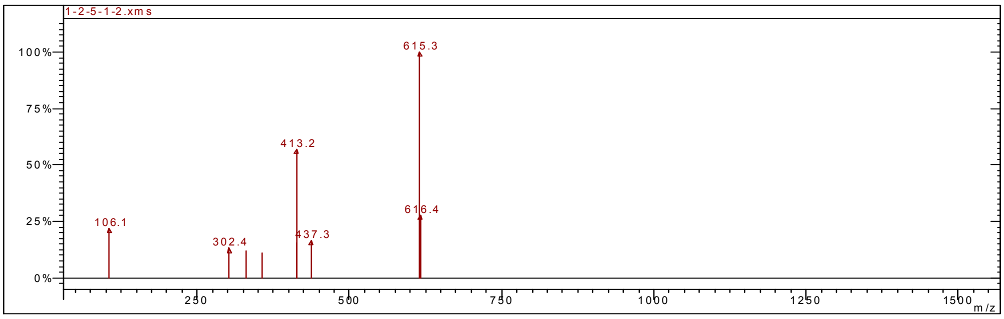

Spectrum from e:\szj\1-2-5-1-2\1-2-5-1-2.xms
Scan No: 301, Time: 5.427 minutes
No averaging. Background corrected.
Comment: 5.427 min. Scan: 301 100.0:1500.0> RIC: 6.624e+7
Pair Count: 9 MW: 0 Formula: None
CAS No: None Acquired Range: 100.0 - 1500.0 m/z

Method Description: ESI
Scan 1 Channel Description: 100.0:1500.0> ESI;CID 175.0;Det 1100;WIQ1
Scan 2 Channel Description: 100.0:1500.0>(-) ESI;CID -120.0;Det 1100;W
Scan Information: cp = 0.1 mTorr
Precursor Mass Range: 100.0 - 1500.0 m/z

Ion	Int	Norm	Ion	Int	Norm	Ion	Int	Norm
106.1	1.573e+6	219	356.3	798325	111	437.3	1.171e+6	163
302.4	942496	132	413.2	4.066e+6	567	615.3	7.160e+6	999
330.2	850787	119	414.3	1.125e+6	157	616.4	1.993e+6	278

(+) ESI

Figure S28. *Cont.*

Scan 306 from e:\szj\1-2-5-1-2\1-2-5-1-2.xms

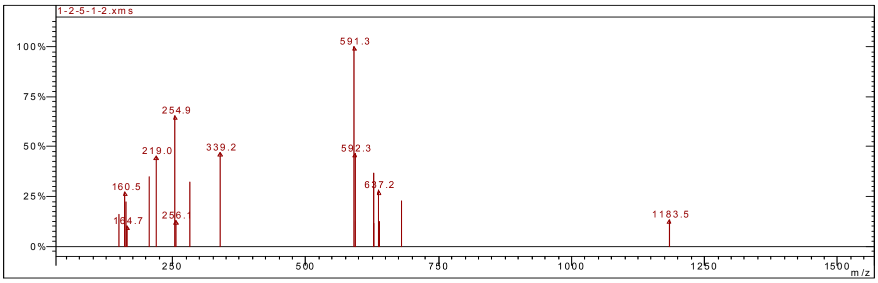

Spectrum from e:\szj\1-2-5-1-2\1-2-5-1-2.xms
Scan No: 306, Time: 5.518 minutes
3 points averaged. Background corrected.
Comment: 5.518 min. Scans: 303-307 100.0:1500.0>(-) RIC: 1.179e+8
Pair Count: 19 MW: 0 Formula: None
CAS No: None Acquired Range: 100.0 - 1500.0 m/z

Method Description: ESI
Scan 1 Channel Description: 100.0:1500.0> ESI;CID 175.0;Det 1100;WIQ1
Scan 2 Channel Description: 100.0:1500.0>(-) ESI;CID -120.0;Det 1100;W
Scan Information: cp = 0.1 mTorr
Precursor Mass Range: 100.0 - 1500.0 m/z

Ion	Int	Norm	Ion	Int	Norm	Ion	Int	Norm
149.1	839841	160	256.1	677556	129	627.3	1.916e+6	365
160.5	1.424e+6	272	282.9	1.687e+6	322	628.3	609348	116
162.5	1.172e+6	224	339.2	2.468e+6	471	637.2	1.466e+6	279
164.7	543761	104	591.3	5.240e+6	999	638.4	655153	125
204.9	1.826e+6	348	592.3	2.447e+6	466	681.2	1.188e+6	227
219.0	2.378e+6	453	593.4	652343	124	1183.5	698558	133
254.9	3.431e+6	654						

(−) ESI

Figure S29. HR-ESI-MS spectrum of compound **3**.

Generic Display Report

Analysis Info

Analysis Name	D:\Data\MS\data\201310\shenzhenxianhu_CYL-1-2-5-1-2_pos.d	Acquisition Date	10/14/2013 8:33:54 AM
Method	POS_100-2000_Dirrect Infusion.m	Operator	SCSIO
Sample Name	SCSIO	Instrument	maXis
Comment			

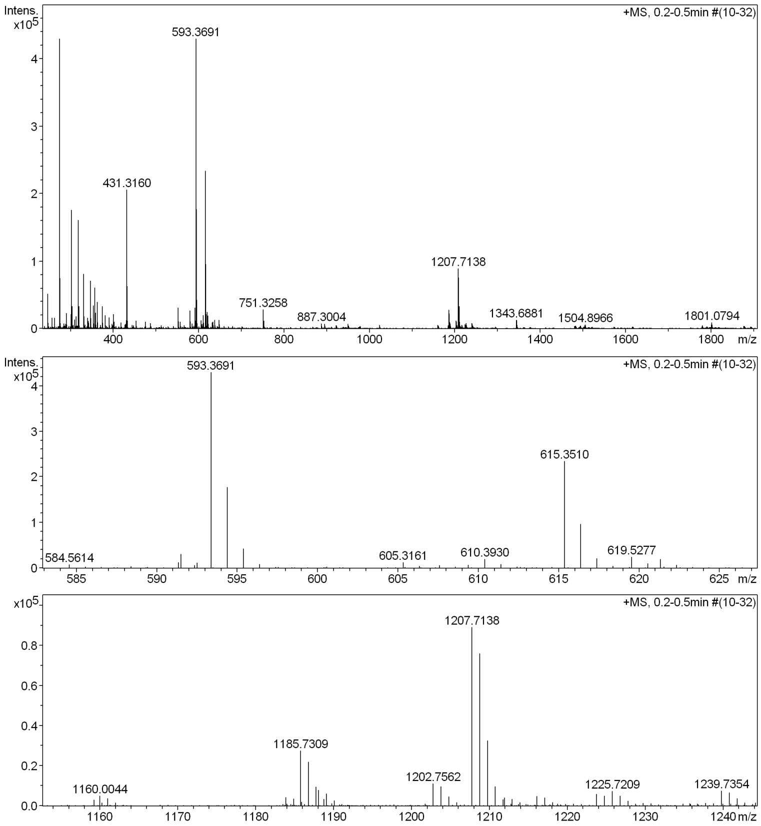

Figure S29. *Cont.*

Mass Spectrum SmartFormula Report

Analysis Info

Analysis Name	D:\Data\MS\data\201310\shenzhenxianhu_CYL-1-2-5-1-2_pos.d	Acquisition Date	10/14/2013 8:33:54 AM
Method	POS_100-2000_Dirrect Infusion.m	Operator	SCSIO
Sample Name	SCSIO	Instrument / Ser#	maXis 29
Comment			

Acquisition Parameter

Source Type	ESI	Ion Polarity	Positive	Set Nebulizer	0.3 Bar
Focus	Not active	Set Capillary	4500 V	Set Dry Heater	180 °C
Scan Begin	100 m/z	Set End Plate Offset	-500 V	Set Dry Gas	4.0 l/min
Scan End	2000 m/z	Set Collision Cell RF	2000.0 Vpp	Set Divert Valve	Waste

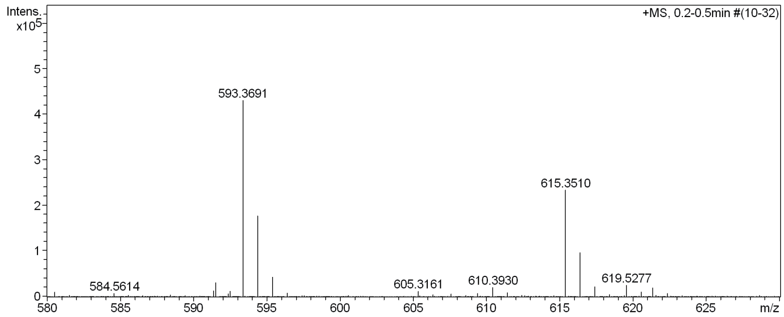

Meas. m/z	#	Formula	Score	m/z	err [mDa]	err [ppm]	mSigma	rdb	e⁻ Conf	N-Rule
593.3691	1	C 33 H 53 O 9	100.00	593.3684	-0.7	-1.1	25.4	7.5	even	ok
615.3510	1	C 33 H 52 Na O 9	100.00	615.3504	-0.7	-1.1	23.9	7.5	even	ok

Bruker Compass DataAnalysis 4.0 printed: 10/14/2013 8:59:50 AM Page 1 of 1

Figure S30. IR spectrum of compound **3**.

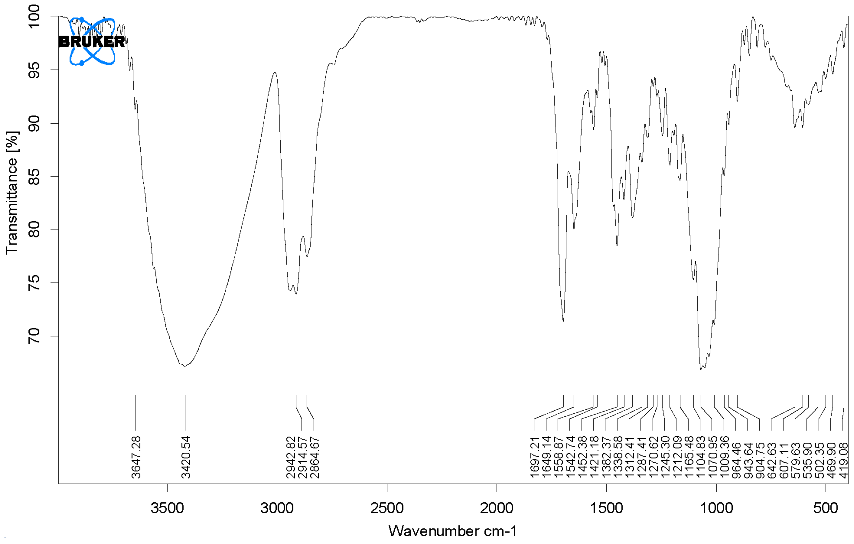

E:\DATABASE\MEAS\ \12512.0 12512 Instrument type and / or accessory

Page 1/1

Figure S31. ^{1}H-NMR spectrum of compound **4**.

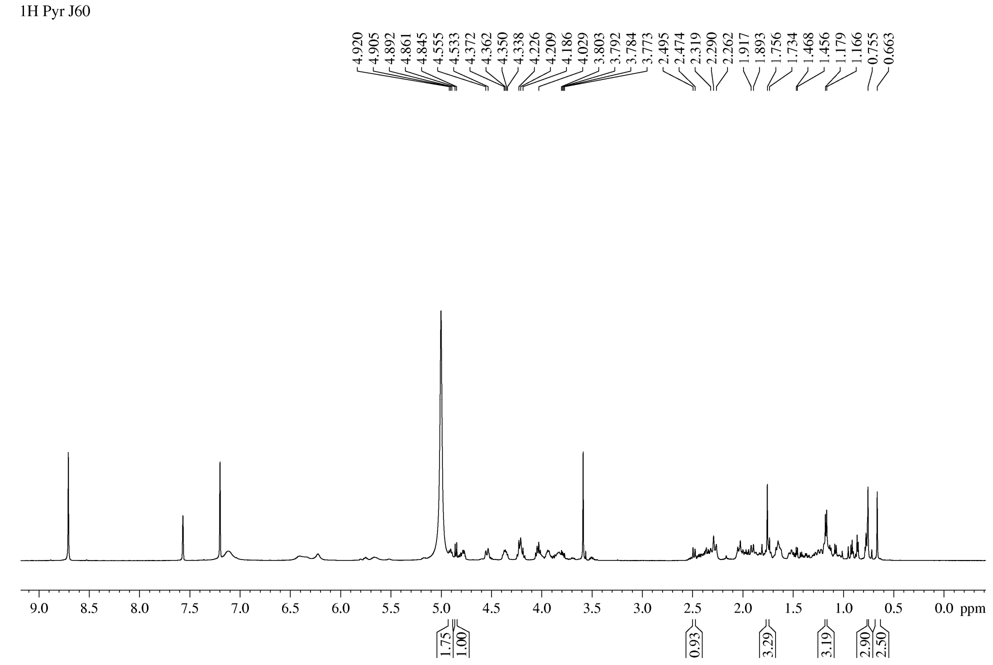

Figure S32. ^{13}C-NMR spectrum of compound **4**.

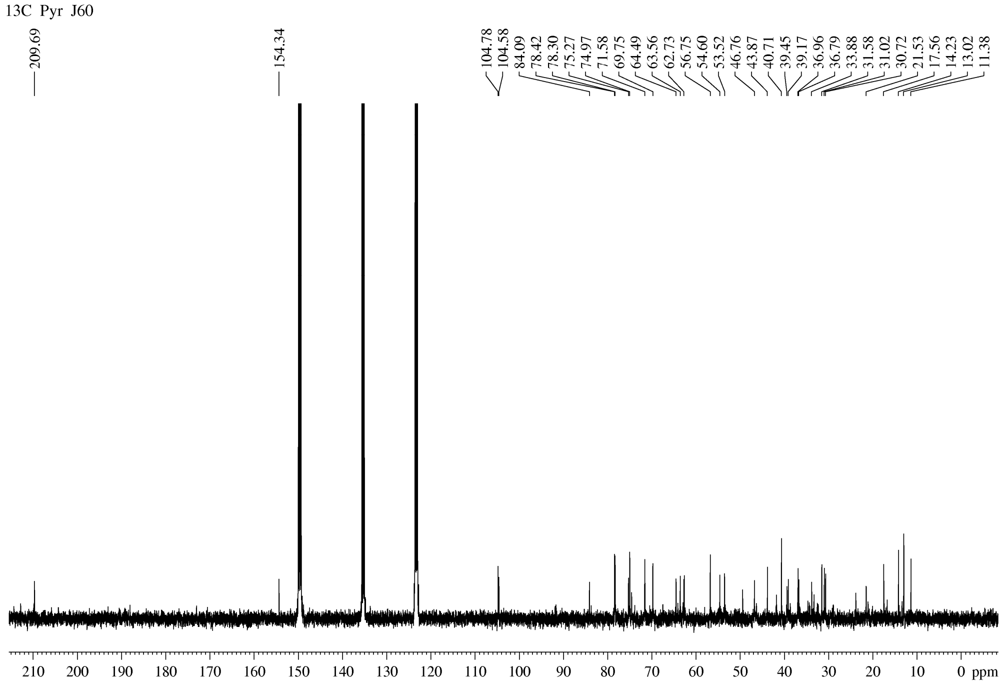

Figure S33. DEPT spectrum of compound **4**.

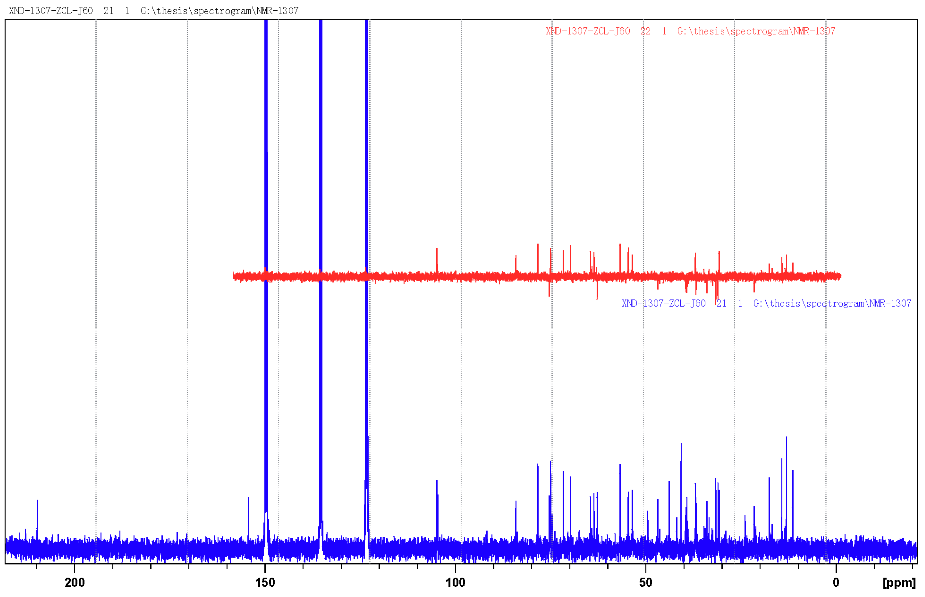

Figure S34. COSY spectrum of compound **4**.

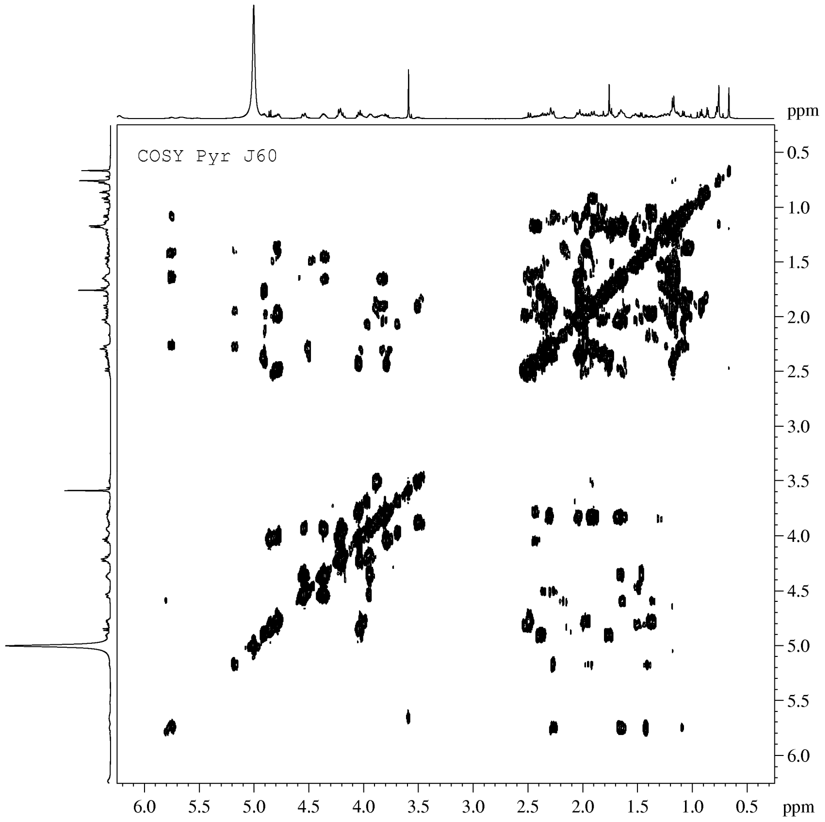

Figure S35. HSQC spectrum of compound **4**.

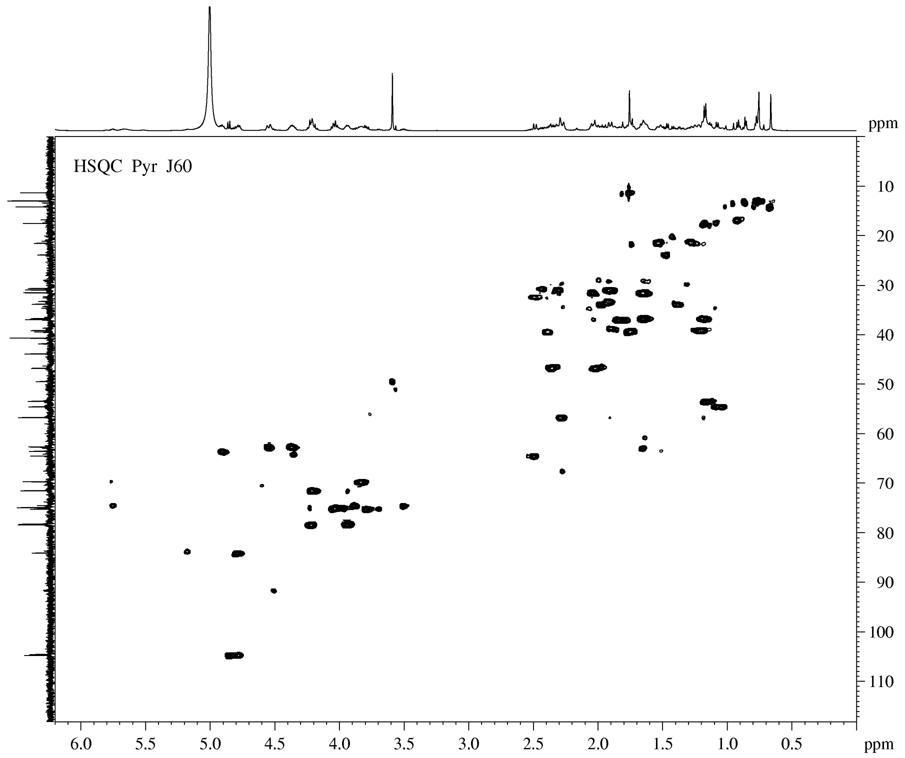

Figure S36. HMBC spectrum of compound **4**.

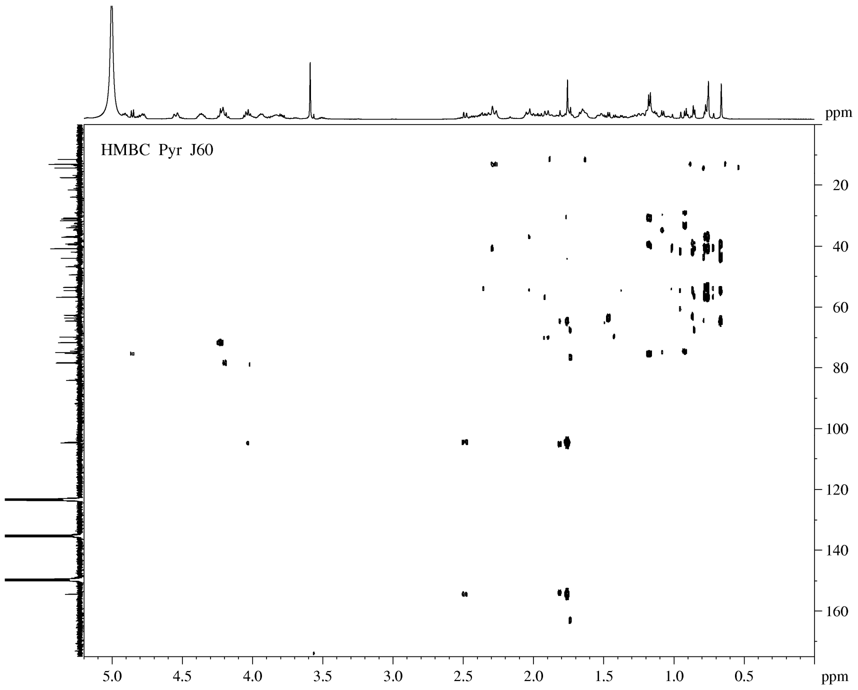

Figure S37. NOESY spectrum of compound **4**.

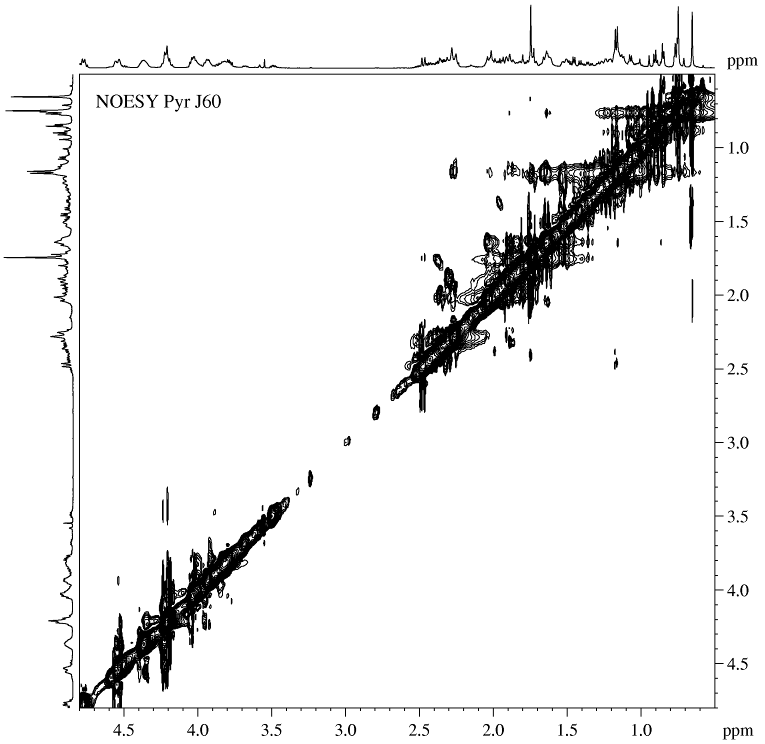

Figure S38. ESI-MS spectrum of compound **4**.

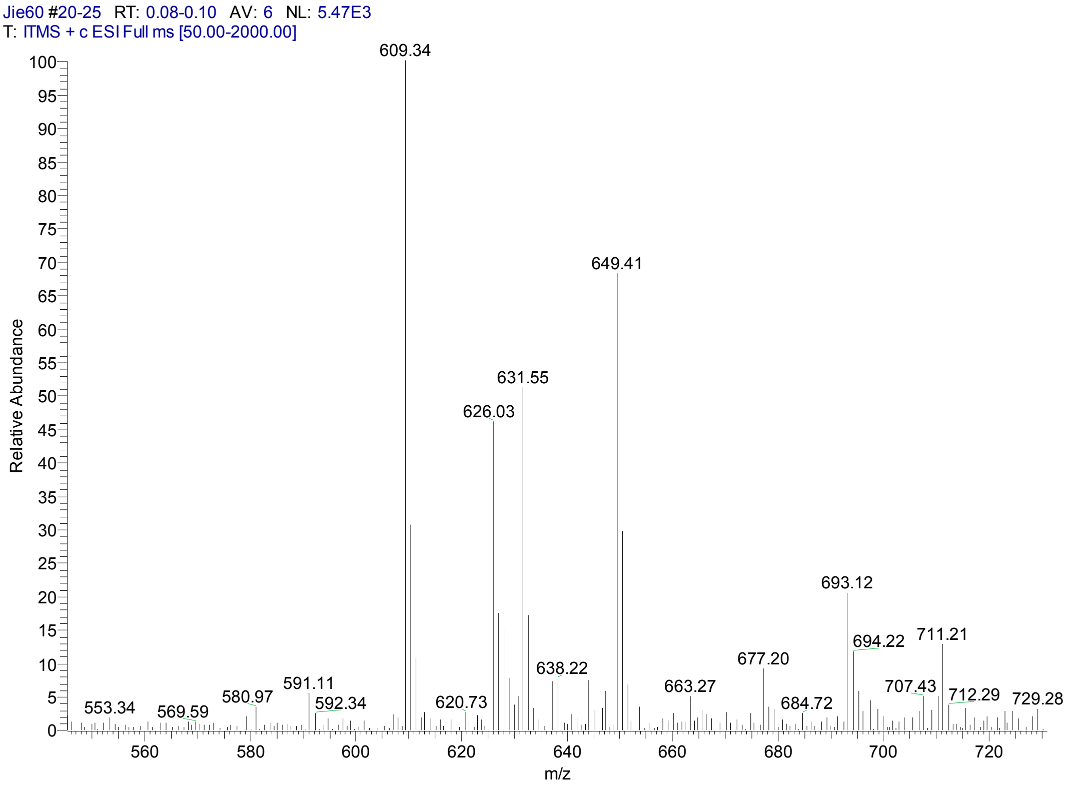

(+) ESI

Figure S38. *Cont.*

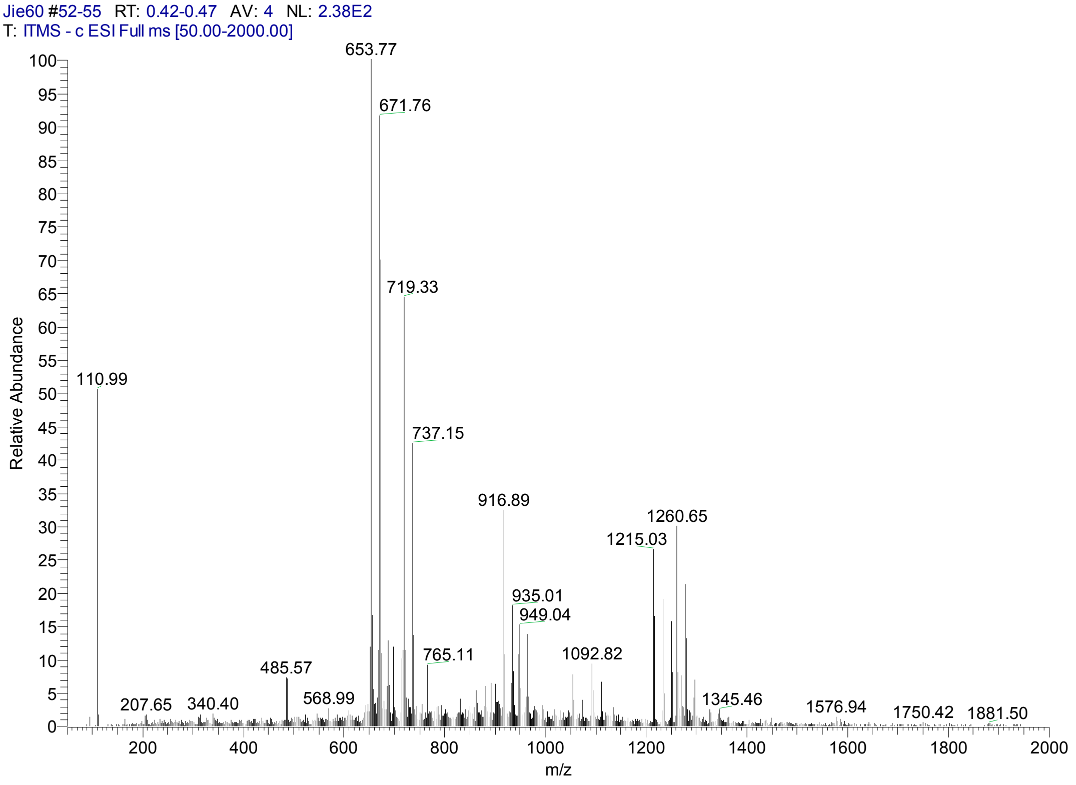

(−) ESI

Figure S39. HR-ESI-MS spectrum of compound **4**.

Mass Spectrum SmartFormula Report

Analysis Info

Analysis Name	D:\Data\MS\data\201312\shenzhenxianhu_J60_pos.d	Acquisition Date	12/23/2013 9:52:52 AM
Method	POS_100-2000_Dirrect Infusion.m	Operator	SCSIO
Sample Name	SCSIO	Instrument / Ser#	maXis 29
Comment			

Acquisition Parameter

Source Type	ESI	Ion Polarity	Positive	Set Nebulizer	0.8 Bar
Focus	Not active	Set Capillary	4800 V	Set Dry Heater	180 °C
Scan Begin	100 m/z	Set End Plate Offset	-500 V	Set Dry Gas	4.0 l/min
Scan End	1500 m/z	Set Collision Cell RF	2000.0 Vpp	Set Divert Valve	Waste

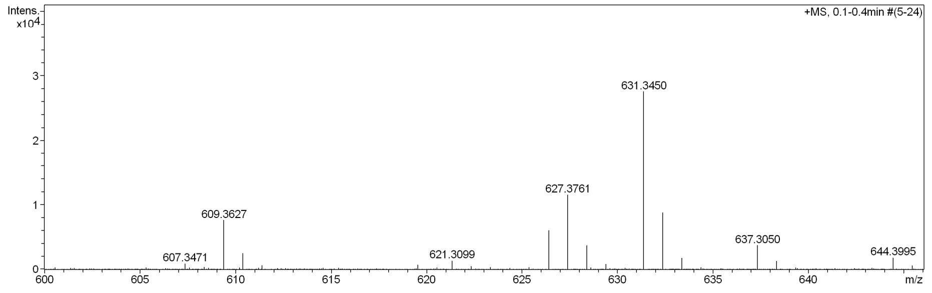

Meas. m/z	#	Formula	Score	m/z	err [mDa]	err [ppm]	mSigma	rdb	e⁻ Conf	N-Rule
609.3627	1	C 33 H 53 O 10	100.00	609.3633	0.7	1.1	19.1	7.5	even	ok
631.3450	1	C 33 H 52 Na O 10	100.00	631.3453	0.3	0.4	26.2	7.5	even	ok

Figure S40. IR spectrum of compound **4**.

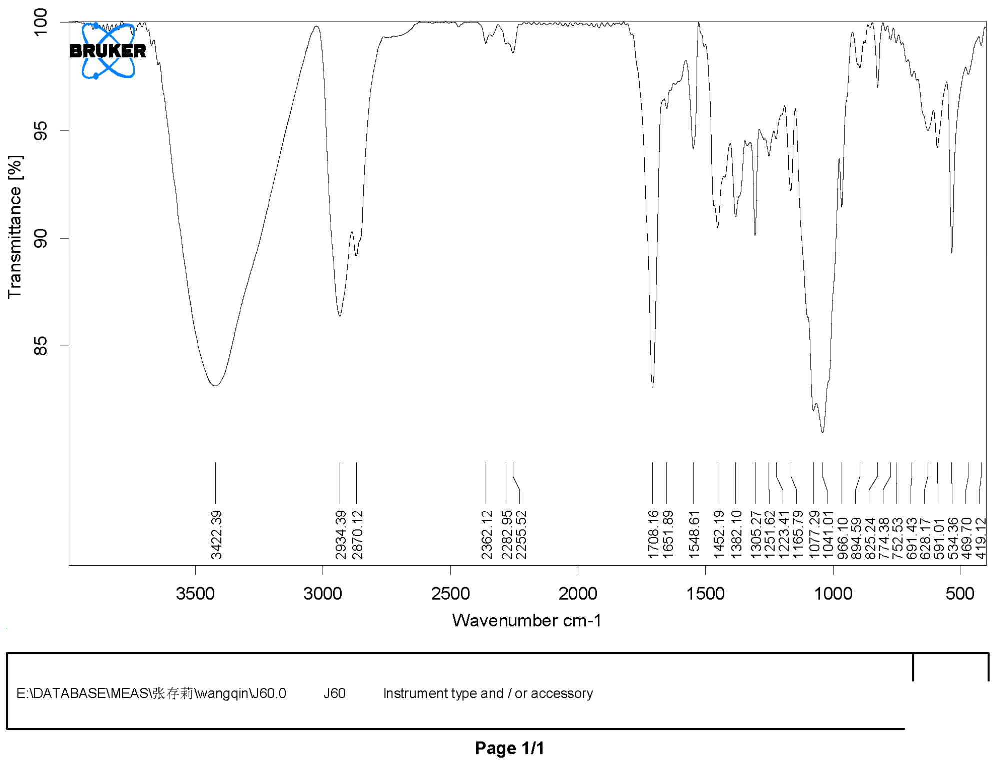

Figure S41. ^{1}H-NMR spectrum of compound **5**.

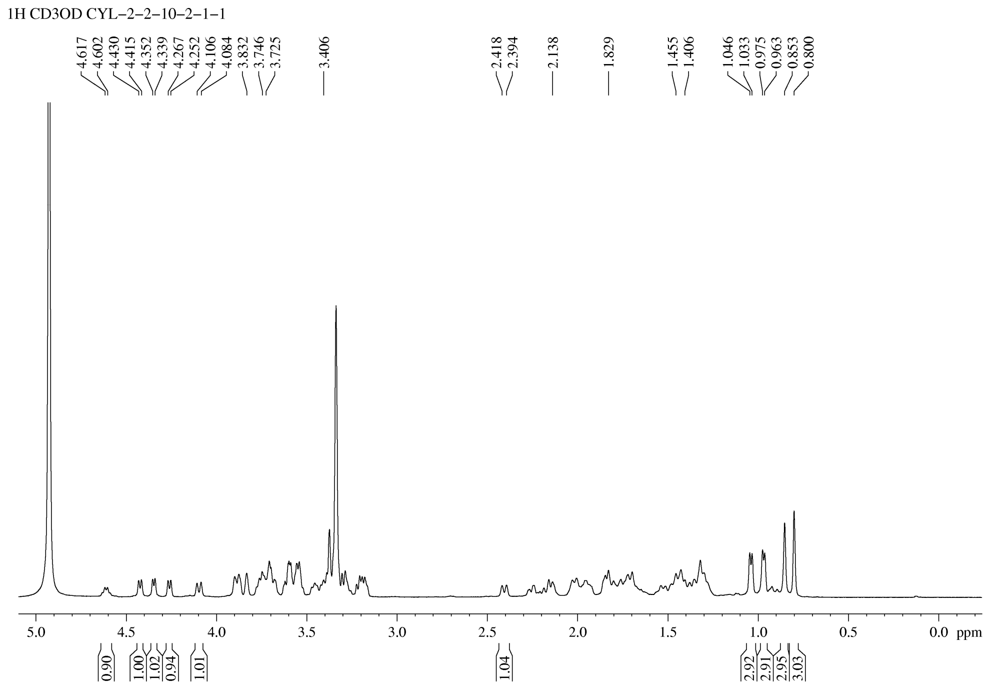

Figure S42. ^{13}C-NMR spectrum of compound **5**.

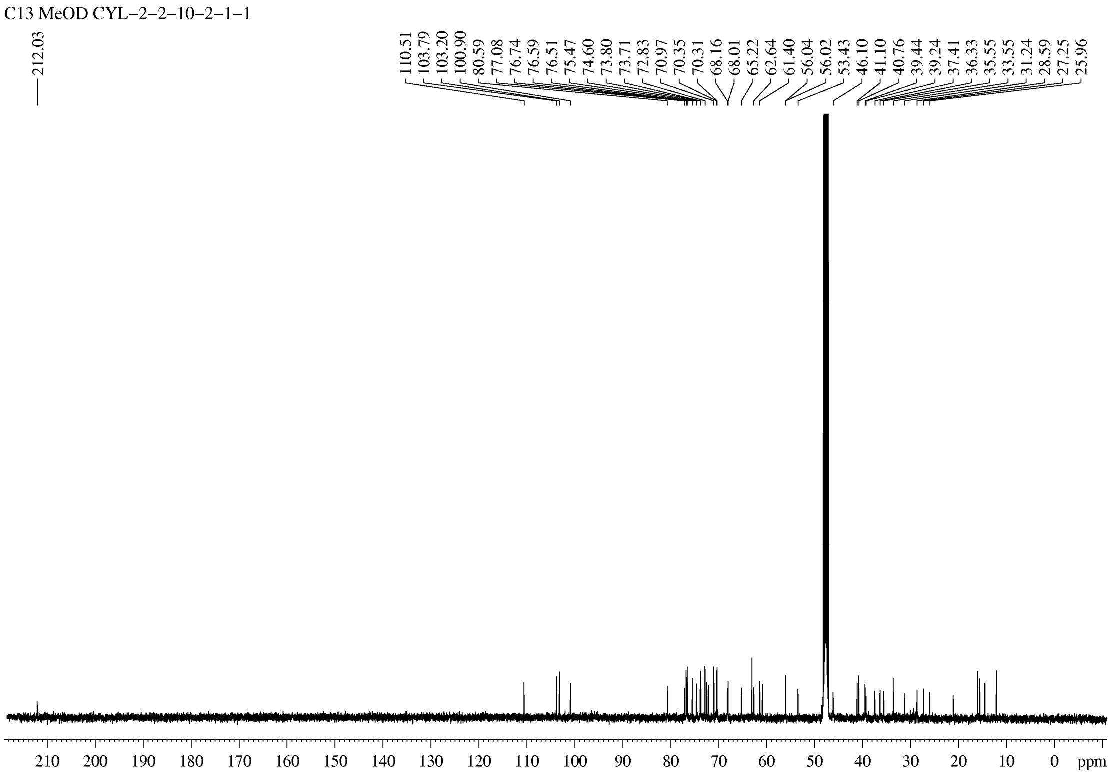

Figure S43. DEPT spectrum of compound **5**.

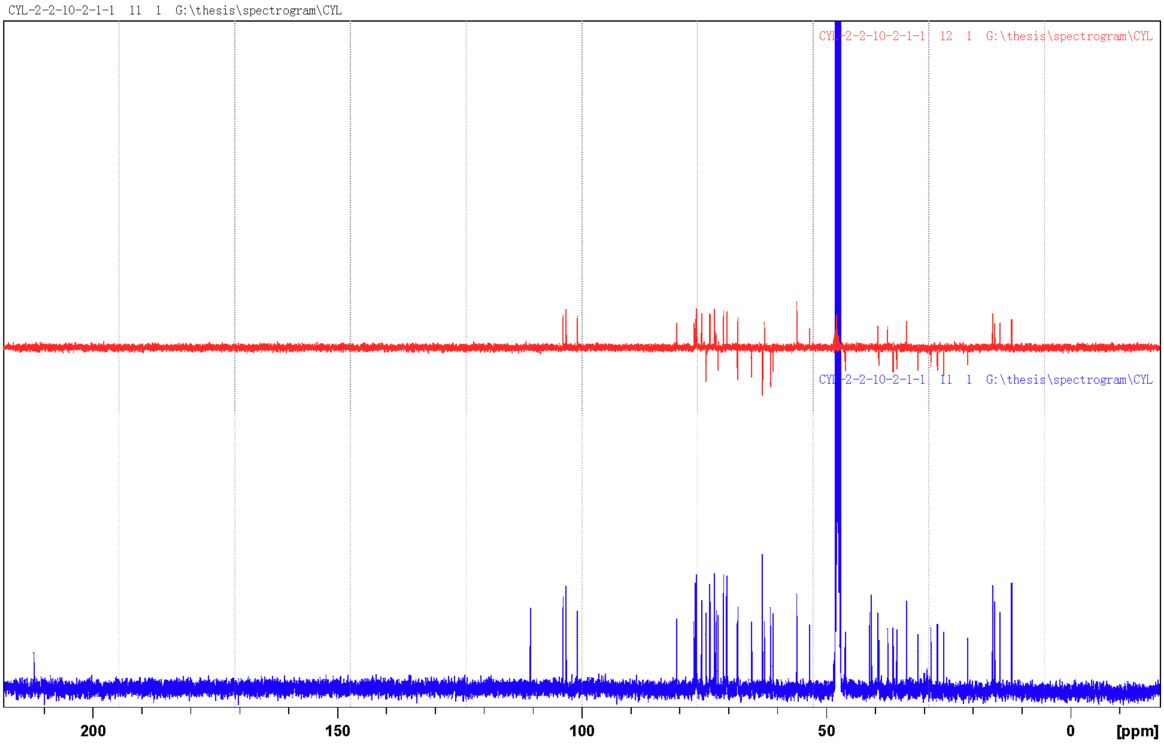

Figure S44. COSY spectrum of compound **5**.

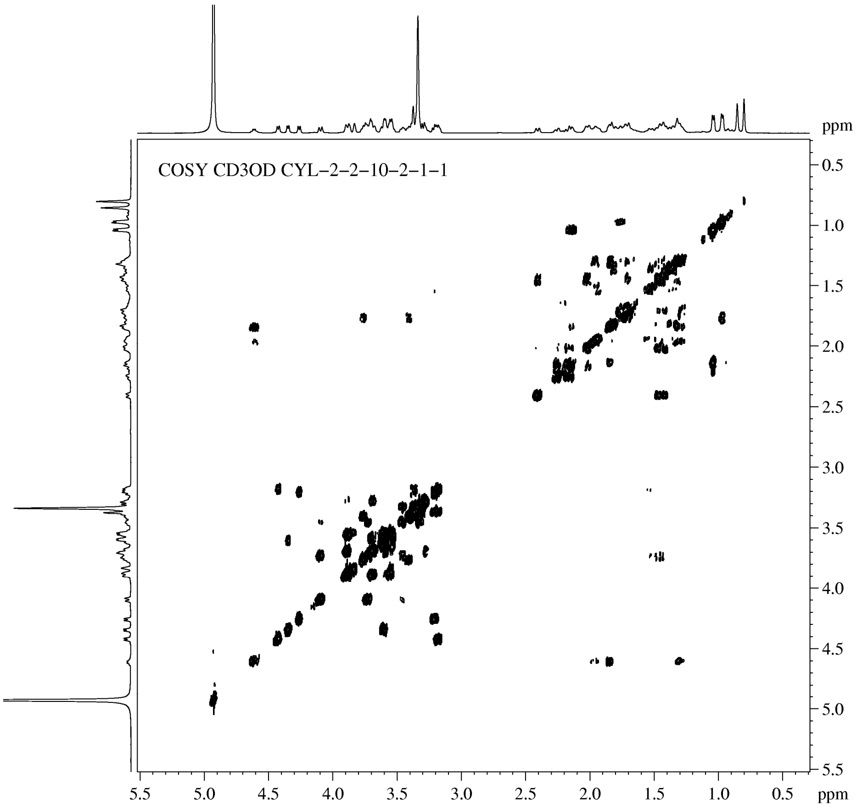

Figure S45. HSQC spectrum of compound **5**.

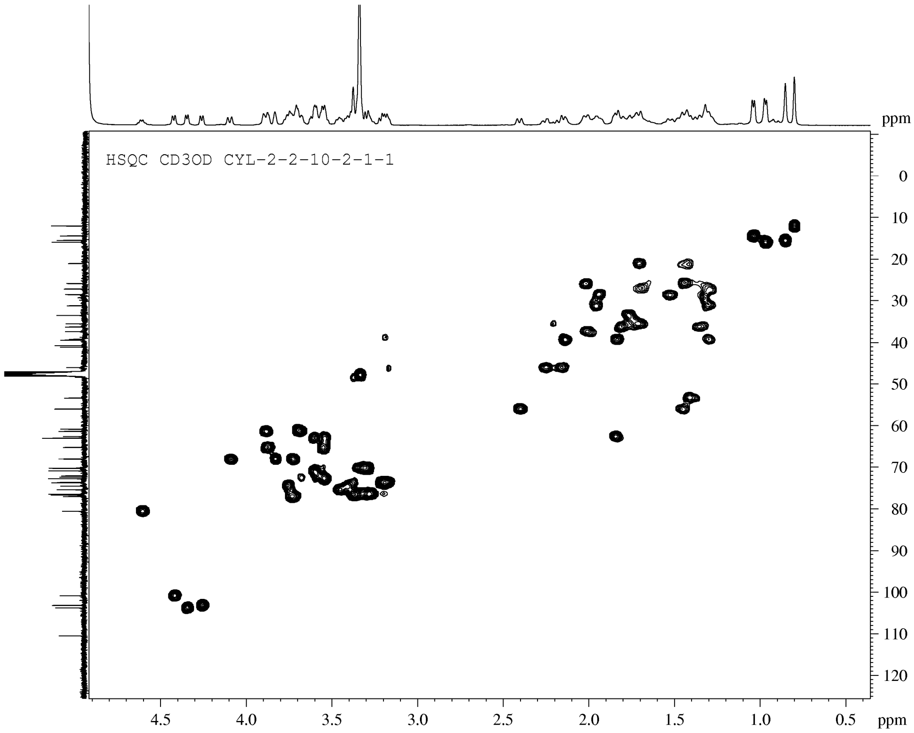

Figure S46. ESI-MS spectrum of compound **5**.

Scan 250 from e:\szj\2-2-10-2-1-1\2-2-10-2-1-1.xms

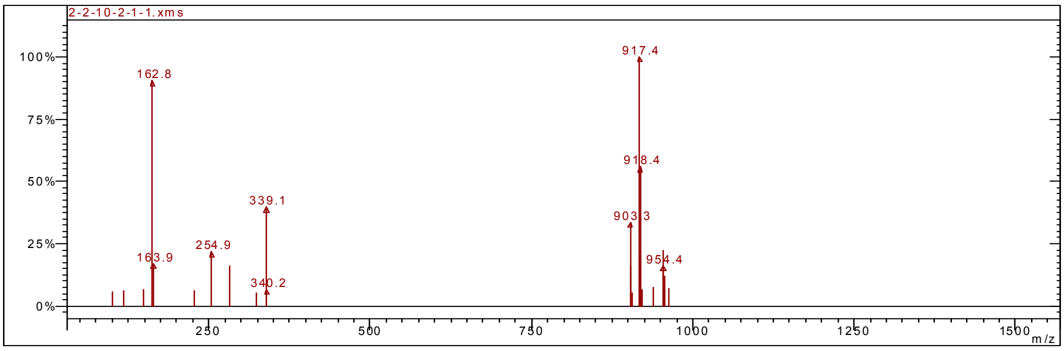

Spectrum from e:\szj\2-2-10-2-1-1\2-2-10-2-1-1.xms
Scan No: 250, Time: 4.505 minutes
5 points averaged. Background corrected.
Comment: 4.505 min. Scans: 245-253 100.0:1500.0>(-) RIC: 2.285e+8
Pair Count: 24 MW: 0 Formula: None
CAS No: None Acquired Range: 100.0 - 1500.0 m/z

Method Description: ESI
Scan 1 Channel Description: 100.0:1500.0> ESI;CID 175.0;Det 1100;W I Q1
Scan 2 Channel Description: 100.0:1500.0>(-) ESI;CID -165.0;Det 1100;W
Scan Information: cp = 0.1 mTorr
Precursor Mass Range: 100.0 - 1500.0 m/z

Ion	Int	Norm	Ion	Int	Norm	Ion	Int	Norm
100.7	964382	56	325.0	916719	54	919.5	3.446e+6	202
119.1	1.061e+6	62	339.1	6.775e+6	396	920.7	1.135e+6	66
148.8	1.108e+6	65	340.2	1.145e+6	67	939.2	1.278e+6	75
162.8	1.543e+7	903	903.3	5.736e+6	335	953.3	3.824e+6	224
163.9	2.884e+6	169	904.4	2.750e+6	161	954.4	2.766e+6	162
226.9	1.022e+6	60	905.4	894434	52	955.4	2.078e+6	122
254.9	3.699e+6	216	917.4	1.708e+7	999	956.3	1.176e+6	69
283.0	2.731e+6	160	918.4	9.534e+6	558	963.3	1.230e+6	72

Figure S47. ^{1}H-NMR spectrum of compound **6**.

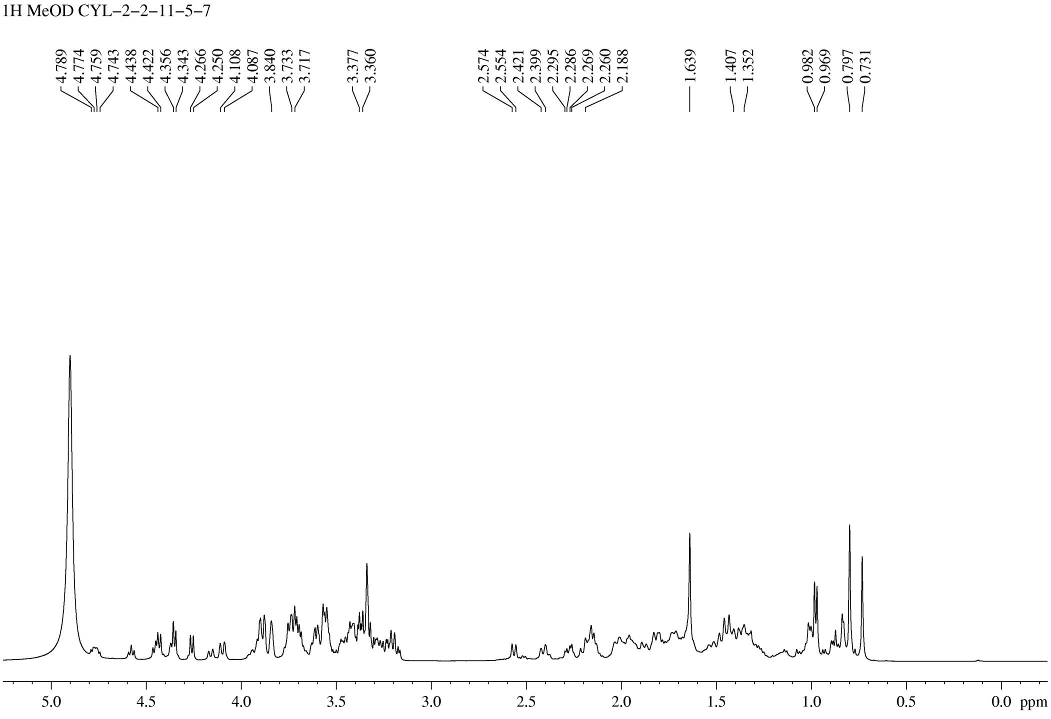

Figure S48. ^{13}C-NMR spectrum of compound **6**.

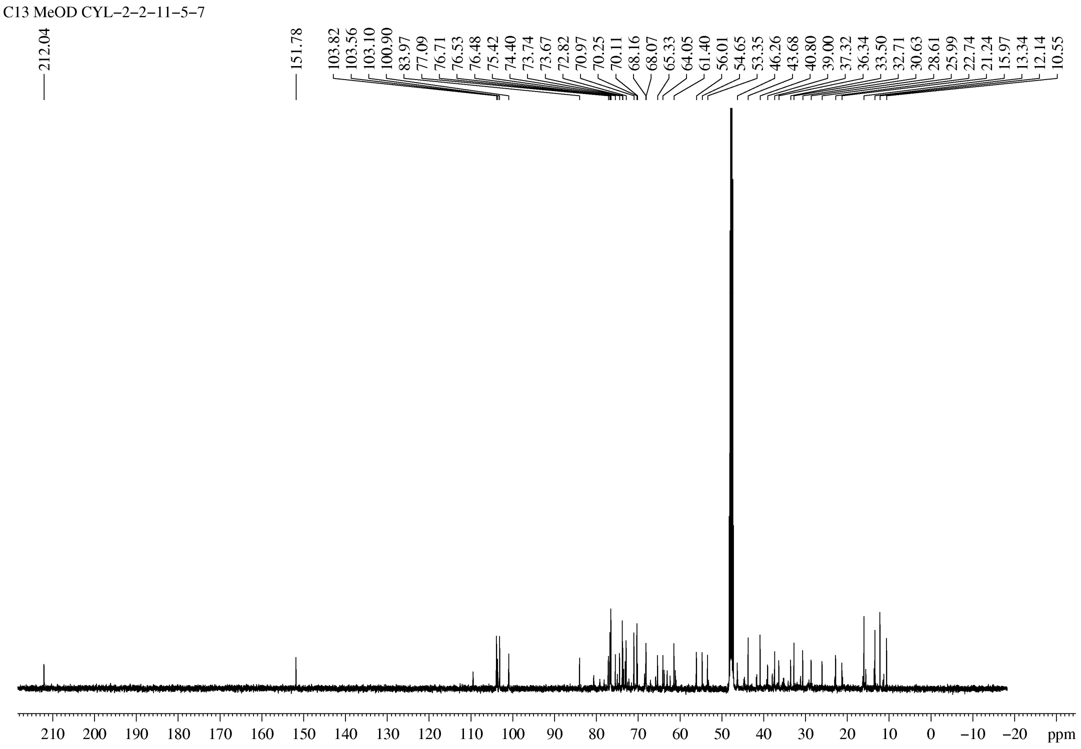

Figure S49. DEPT spectrum of compound **6**.

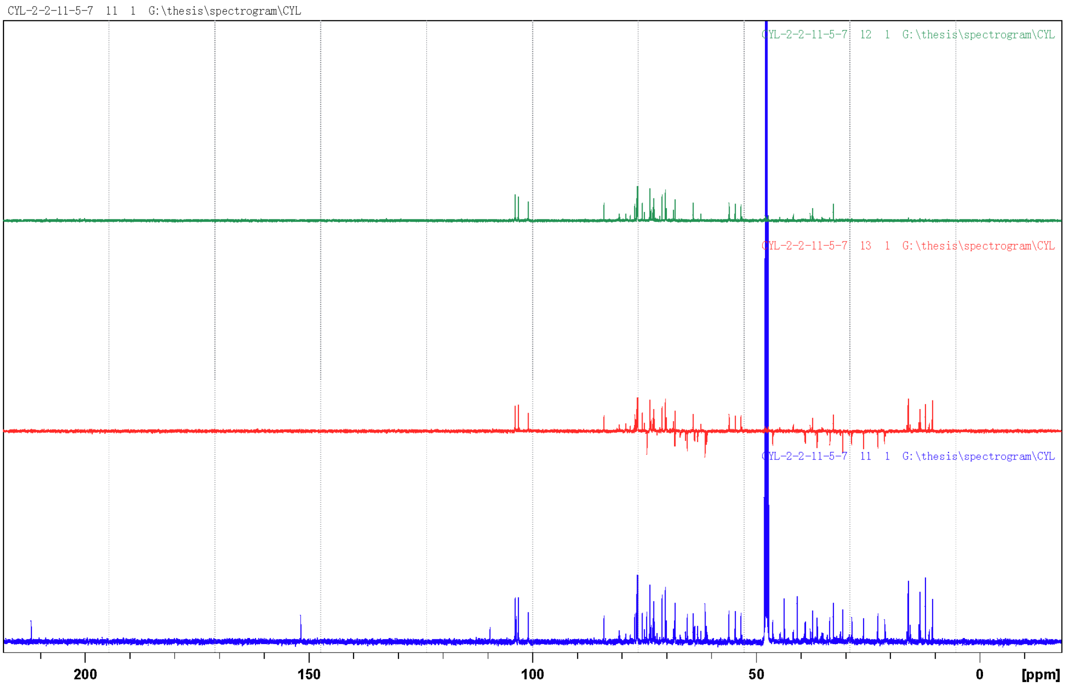

Figure S50. HSQC spectrum of compound **6**.

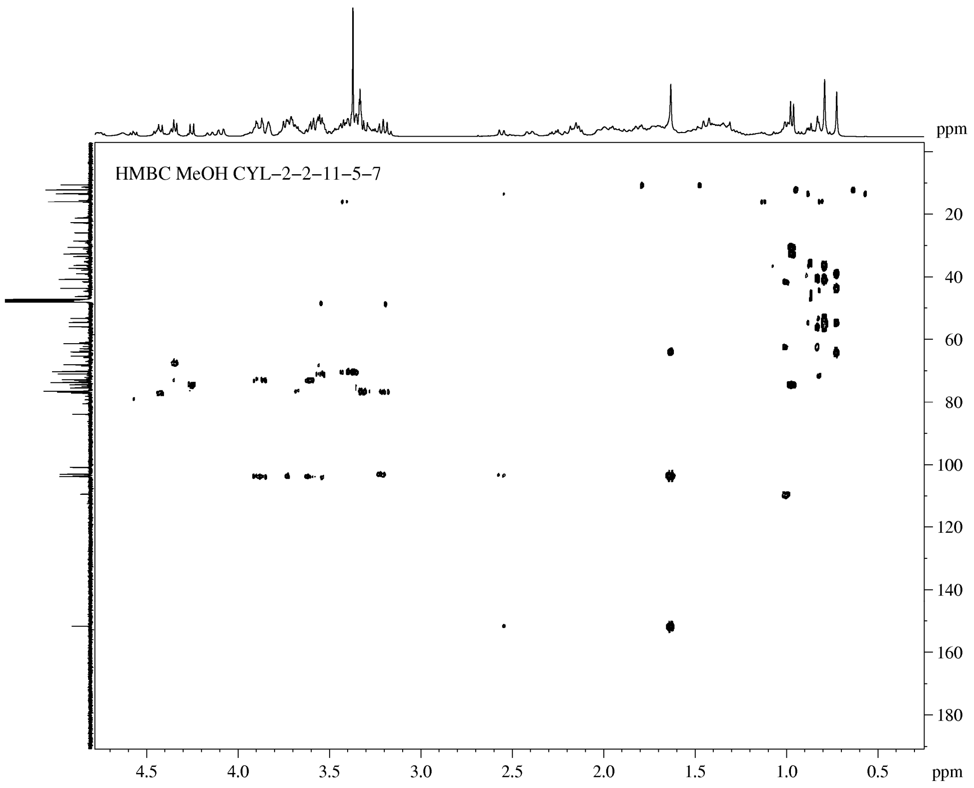

Figure S51. HMBC spectrum of compound **6**.

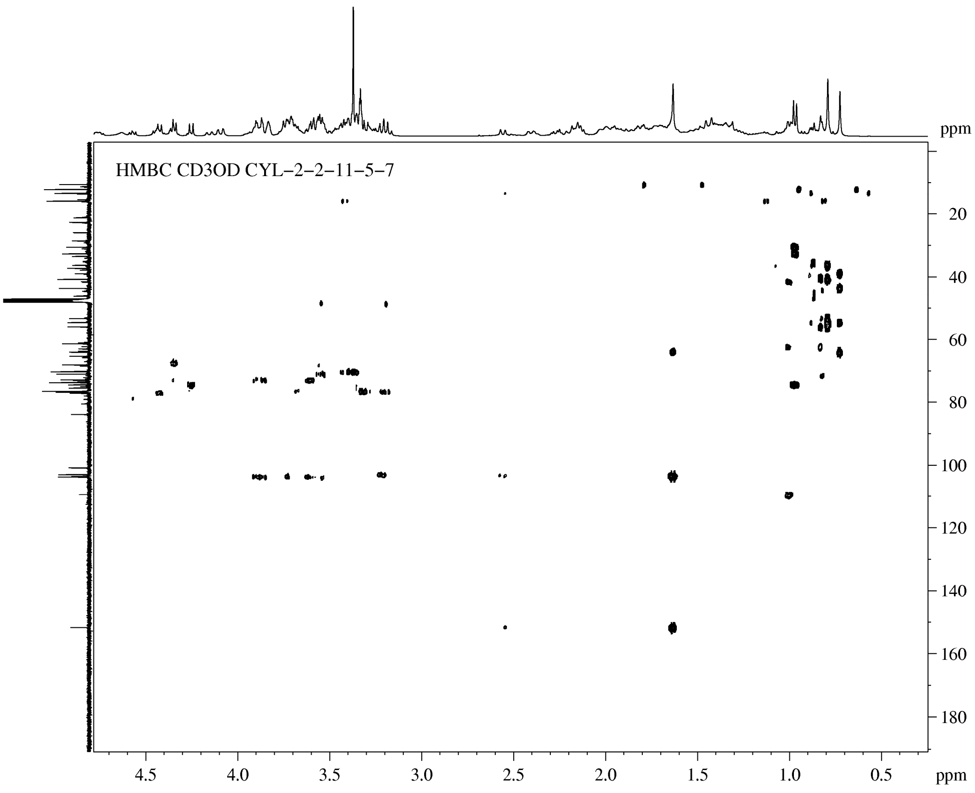

Figure S52. ESI-MS spectrum of compound **6**.

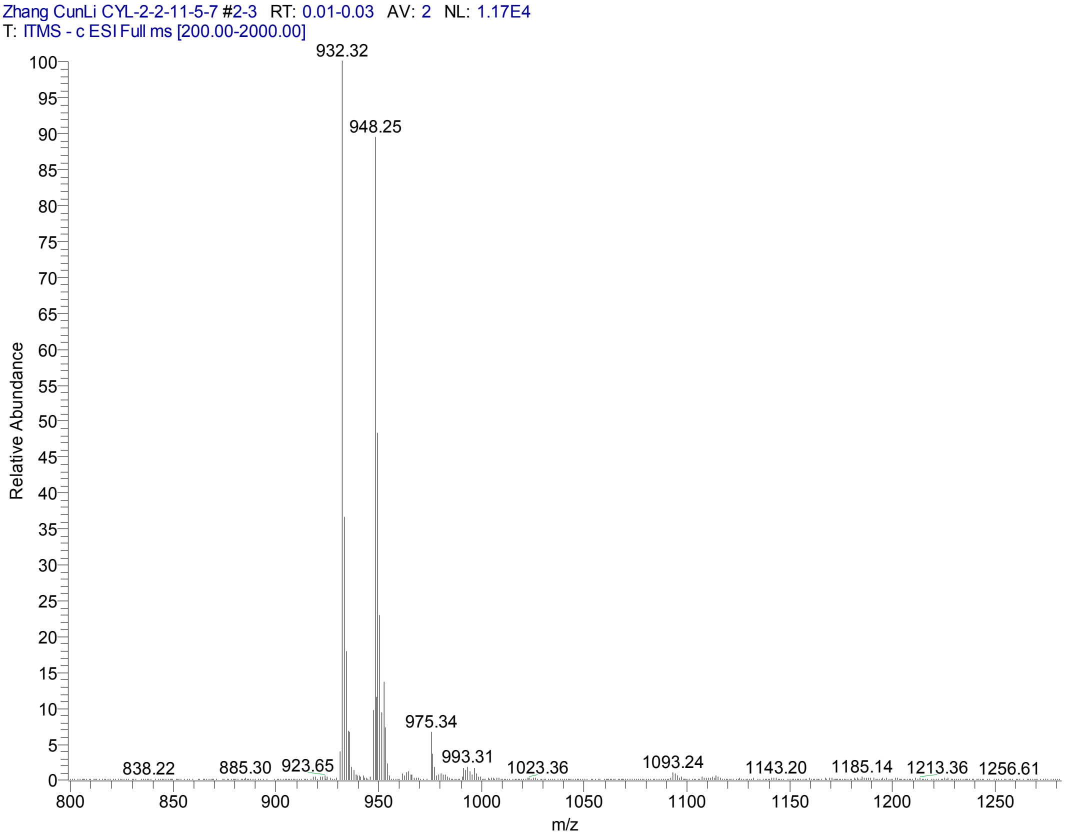

Figure S53. ^{1}H-NMR spectrum of compound **7**.

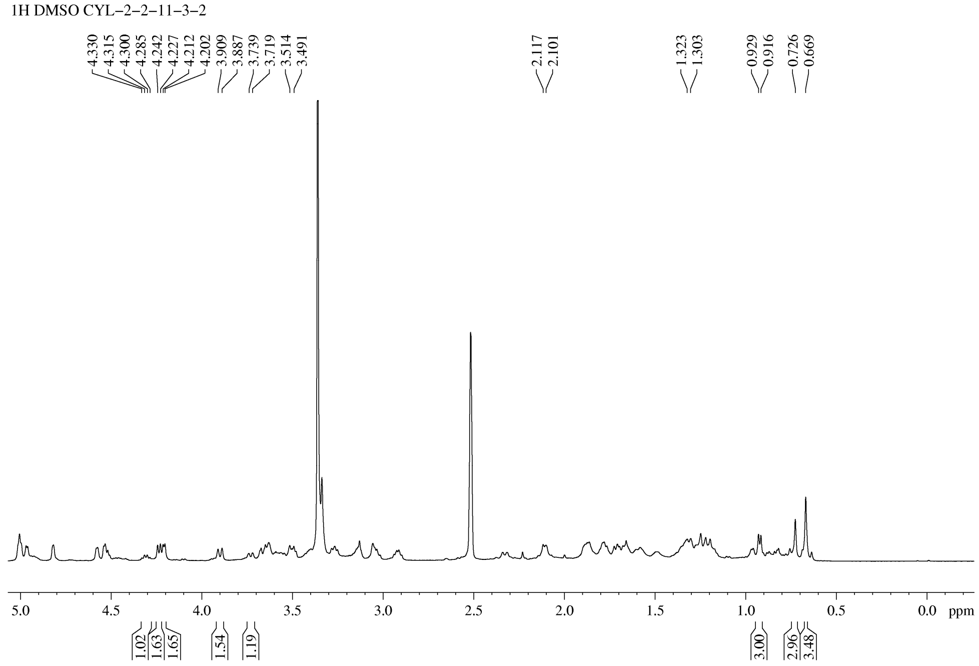

Figure S54. ^{13}C-NMR spectrum of compound **7**.

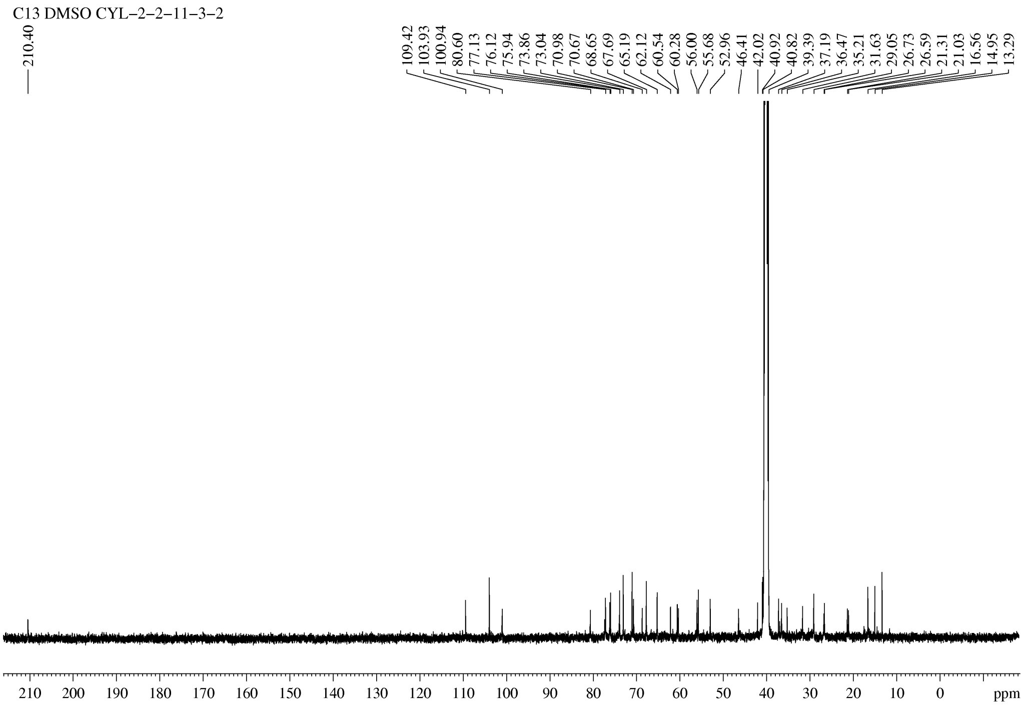

Figure S55. DEPT spectrum of compound **7**.

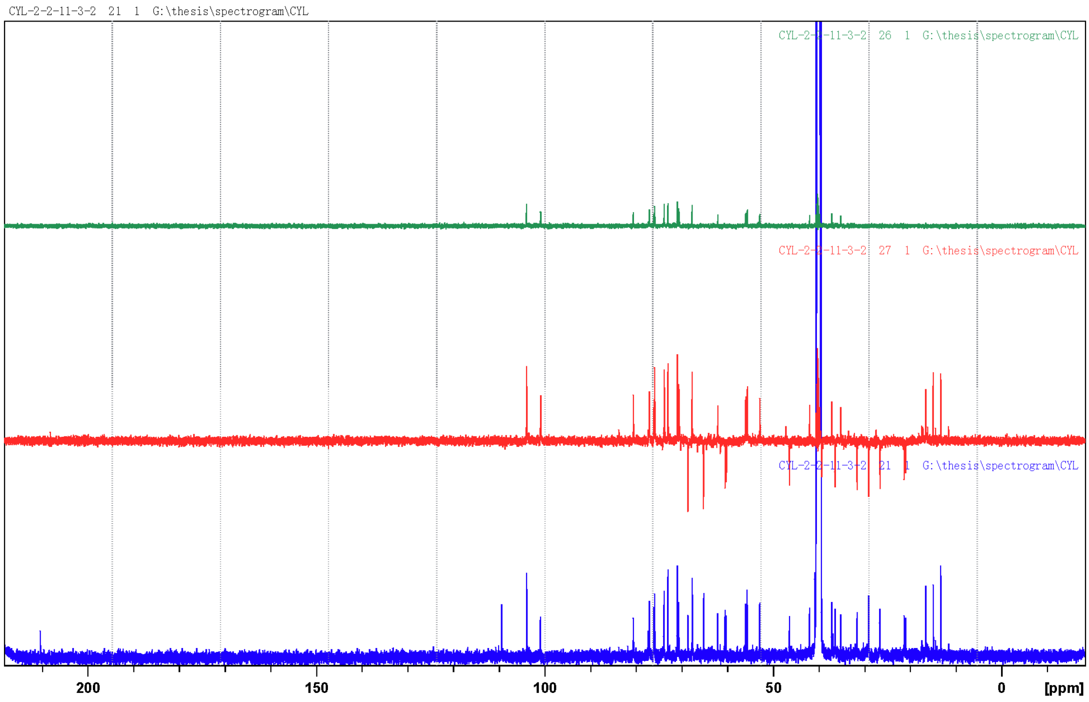

Figure S56. HSQC spectrum of compound **7**.

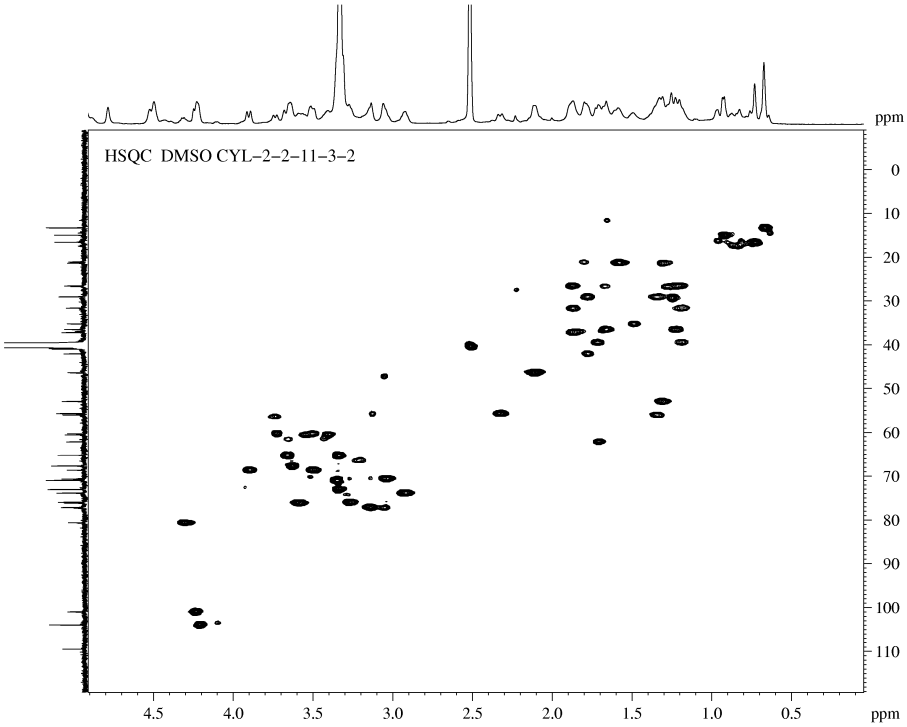

Supplement: Supplementary file 1 [file molecules-19-20975-s001.pdf]
